# Preparation of High-Purity Ammonium Tetrakis(pentafluorophenyl)borate for the Activation of Olefin Polymerization Catalysts

**DOI:** 10.3390/molecules26092827

**Published:** 2021-05-10

**Authors:** Hyun-Ju Lee, Jun-Won Baek, Yeong-Hyun Seo, Hong-Cheol Lee, Sun-Mi Jeong, Junseong Lee, Chong-Gu Lee, Bun-Yeoul Lee

**Affiliations:** 1 Department of Molecular Science and Technology, Ajou University, Suwon 16499, Korea; hjulee4639@ajou.ac.kr (H.-J.L.); btw91@ajou.ac.kr (J.-W.B.); tdg0730@ajou.ac.kr (Y.-H.S.); asdfg7235950@gmail.com (H.-C.L.); sunmi7523@ajou.ac.kr (S.-M.J.); 2 Department of Chemistry, Chonnam National University, 77 Yongbong-ro, Buk-gu, Gwangju 61186, Korea; leespy@chonnam.ac.kr; 3 Precious Catalysts Inc., 201 Duryu-gil, Angangeup, Gyeongju 38029, Korea; cglee@s-pci.com

**Keywords:** metallocene, half-metallocene, activation reaction, ammonium tetrakis(pentafluorophenyl)borate, olefin polymerization

## Abstract

Homogeneous olefin polymerization catalysts are activated in situ with a co-catalyst ([PhN(Me)_2_-H]^+^[B(C_6_F_5_)_4_]^−^ or [Ph_3_C]^+^[B(C_6_F_5_)_4_]^−^) in bulk polymerization media. These co-catalysts are insoluble in hydrocarbon solvents, requiring excess co-catalyst (>3 eq.). Feeding the activated species as a solution in an aliphatic hydrocarbon solvent may be advantageous over the in situ activation method. In this study, highly pure and soluble ammonium tetrakis(pentafluorophenyl)borates ([Me(C_18_H_37_)_2_N-H]^+^[B(C_6_F_5_)_4_]^−^ and [(C_18_H_37_)_2_NH_2_]^+^[B(C_6_F_5_)_4_]^−^) containing neither water nor Cl^−^ salt impurities were prepared easily via the acid–base reaction of [PhN(Me)_2_-H]^+^[B(C_6_F_5_)_4_]^−^ and the corresponding amine. Using the prepared ammonium salts, the activation reactions of commercial-process-relevant metallocene (*rac*-[ethylenebis(tetrahydroindenyl)]Zr(Me)_2_ (**1**-ZrMe_2_), [Ph_2_C(Cp)(3,6-*^t^*Bu_2_Flu)]Hf(Me)_2_ (**3**-HfMe_2_), [Ph_2_C(Cp)(2,7-*^t^*Bu_2_Flu)]Hf(Me)_2_ (**4**-HfMe_2_)) and half-metallocene complexes ([(η^5^-Me_4_C_5_)Si(Me)_2_(κ-N*^t^*Bu)]Ti(Me)_2_ (**5**-TiMe_2_), [(η^5^-Me_4_C_5_)(C_9_H_9_(κ-N))]Ti(Me)_2_ (**6**-TiMe_2_), and [(η^5^-Me_3_C_7_H_1_S)(C_10_H_11_(κ-N))]Ti(Me)_2_ (**7**-TiMe_2_)) were monitored in C_6_D_12_ with ^1^H NMR spectroscopy. Stable [**L**-M(Me)(NMe(C_18_H_37_)_2_)]^+^[B(C_6_F_5_)_4_]^−^ species were cleanly generated from **1**-ZrMe_2_, **3**-HfMe_2_, and **4**-HfMe_2_, while the species types generated from **5**-TiMe_2_, **6**-TiMe_2_, and **7**-TiMe_2_ were unstable for subsequent transformation to other species (presumably, [**L**-Ti(CH_2_N(C_18_H_37_)_2_)]^+^[B(C_6_F_5_)_4_]^−^-type species). [**L**-TiCl(N(H)(C_18_H_37_)_2_)]^+^[B(C_6_F_5_)_4_]^−^-type species were also prepared from **5**-TiCl(Me) and **6**-TiCl(Me), which were newly prepared in this study. The prepared [**L**-M(Me)(NMe(C_18_H_37_)_2_)]^+^[B(C_6_F_5_)_4_]^−^-, [**L**-Ti(CH_2_N(C_18_H_37_)_2_)]^+^[B(C_6_F_5_)_4_]^−^-, and [**L**-TiCl(N(H)(C_18_H_37_)_2_)]^+^[B(C_6_F_5_)_4_]^−^-type species, which are soluble and stable in aliphatic hydrocarbon solvents, were highly active in ethylene/1-octene copolymerization performed in aliphatic hydrocarbon solvents.

## 1. Introduction

Since the serendipitous discovery of methylaluminoxane (MAO) by Kaminsky, much effort has been devoted to the synthesis of organometallic complexes to find efficient single-site homogeneous olefin polymerization catalysts, some of which are currently used in the bulk polyolefin industry [1]. The initial metallocene complexes (i.e., **L**-MCl_2_-type complexes, where L and M are cyclopentadienyl-type ligands and group-four metals, respectively) have been followed by the development of half-metallocene complexes, in which one of the two cyclopentadienyl-type ligands in metallocenes is replaced with an amido or aryloxo ligand, and further by post-metallocene complexes that do not contain any cyclopentadienyl-type ligands [2,3]. The central group-four transition metals have also been expanded to Ni, Pd, Fe, Co, Cr, and V, especially in post-metallocene complexes [4,5,6,7]. Metallocene, half-metallocene, and post-metallocene complexes are not active, and must be activated with a co-catalyst. MAO is a typical example of a co-catalyst used in activation reactions. The structure of MAO (obtained through partial hydrolysis of Me_3_Al) is ill-defined, but may be oligomeric Me_2_Al-[Al(Me)O]_n-_AlMe_2_, containing some free Me_3_Al, and the mechanism of the activation reaction with MAO is still elusive [8,9,10]. In industry, MAO-activated catalysts are used mainly in a slurry or gas phase process after anchoring on the surface of silica particles, where morphological control of the generated polymer particles is crucial [11,12]. MAO is expensive and should be fed in excess relative to organometallic complexes (Al/M > 100). In a slurry or gas phase process, the catalyst retention time is long (>1 h) and the productivity of the catalyst can be maximized, mitigating the burden of the catalyst and co-catalyst cost.

**Scheme 1:**
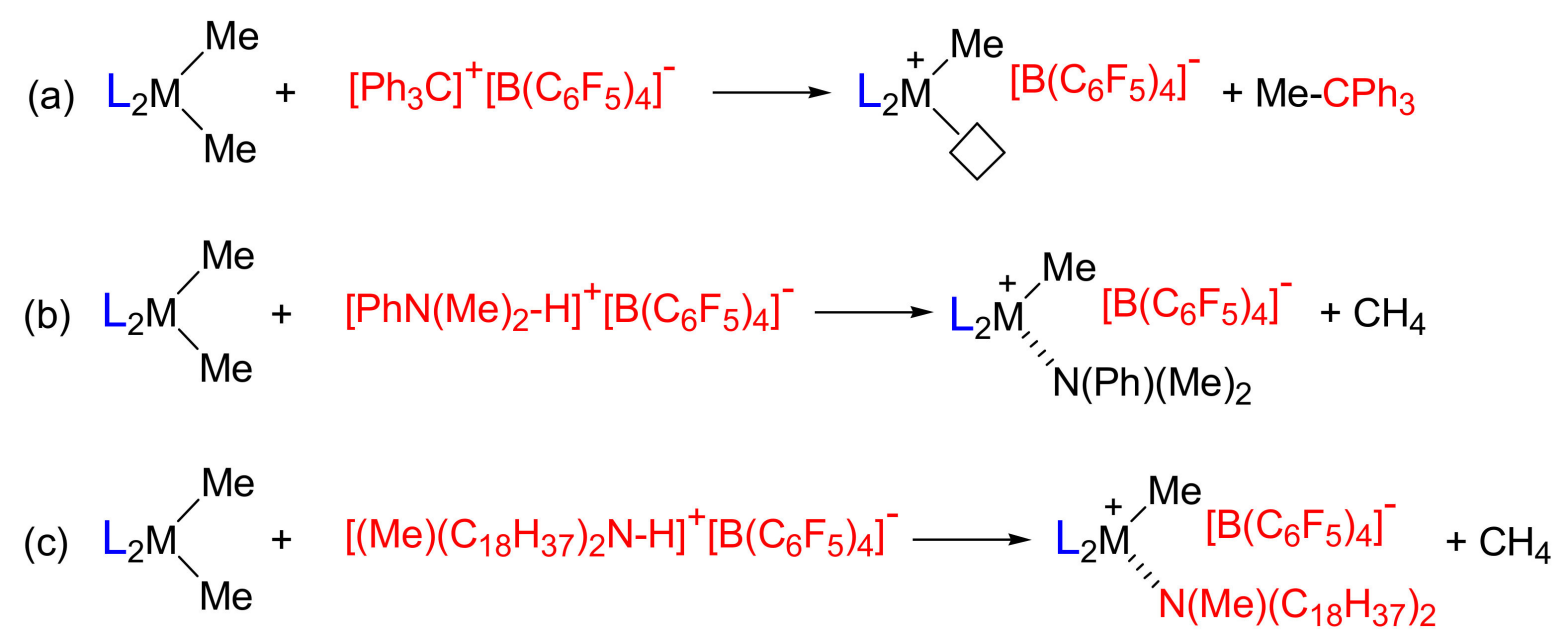
Activation reactions of organometallic complexes for olefin polymerization with [Ph_3_C]^+^[B(C_6_F_5_)_4_]^−^ (**a**), [PhN(Me)_2_-H]^+^[B(C_6_F_5_)_4_]^−^ (**b**), and [Me(C_18_H_37_)_2_N-H]^+^[B(C_6_F_5_)_4_]^−^ (**c**).

Another type of co-catalyst, [PhN(Me)_2_-H]^+^[B(C_6_F_5_)_4_]^−^ or [Ph_3_C]^+^[B(C_6_F_5_)_4_]^−^, was developed for the activation of organometallic complexes, which is used in a solution process in which the catalyst retention time is relatively short (<20 min) [13,14,15]. Replacement of excess MAO with stoichiometric amounts of [B(C_6_F_5_)_4_]^−^-based co-catalyst has also been a crucial issue in the development of Cr-based ethylene tetramerization catalysts [16,17,18]. In the activation reaction, **L**-M(Me)_2_-type complexes were converted to an ion-pair complex [**L**-M(Me)]^+^[B(C_6_F_5_)_4_]^−^ by the action of the co-catalyst (Scheme 1a–c) [19,20,21]. [PhN(Me)_2_-H]^+^[B(C_6_F_5_)_4_]^−^ and [Ph_3_C]^+^[B(C_6_F_5_)_4_]^−^ are well-defined discrete molecules and, conceptually, stoichiometric (equimolar) amounts can be used for the activation reaction, reducing the burden of co-catalyst cost even in a solution process with a short retention time. However, [PhN(Me)_2_-H]^+^[B(C_6_F_5_)_4_]^−^ and [Ph_3_C]^+^[B(C_6_F_5_)_4_]^−^ are insoluble in aliphatic hydrocarbon solvents (e.g., hexane, cyclohexane, or methylcyclohexane), in which the commercial solution process of olefin polymerization is performed. The poor solubility of these co-catalysts causes a burden in some cases, and synthesis of the soluble form has been pursued [22,23,24]. Typically, in order to achieve optimal productivity, an excess amount of [B(C_6_F_5_)_4_]^−^-based co-catalyst (>3 equiv.) should be fed as a slurry phase directly into a bulk polymerization reactor in which both the activation reaction and polymerization reactions take place [25]. After activation in a more polar aromatic hydrocarbon solvent (e.g., toluene), the activated complex may be fed into a reactor filled with aliphatic hydrocarbon solvent. However, [PhN(Me)_2_-H]^+^[B(C_6_F_5_)_4_]^−^ and [Ph_3_C]^+^[B(C_6_F_5_)_4_]^−^ are either completely insoluble or sparingly soluble, respectively, in toluene [26], which still requires an excess amount (e.g., 4 equiv.) to achieve optimal productivity [27,28,29]. For a typical post-metallocene pyridylamido-Hf complex, the activation reaction was thoroughly investigated, and was more complicated than previously thought [30,31,32]. Trialkylaluminum has been additionally fed into the activation reaction to scrub the impurities and to convert **L**-MCl_2_-type complexes into **L**-MR_2_, but [Ph_3_C]^+^[B(C_6_F_5_)_4_]^−^ is destroyed by the action of trialkylaluminum, making the activation reaction more tricky [33].

The best strategy may be the generation of the activated complex in an aliphatic hydrocarbon solvent and feeding of the solution containing the activated species to a bulk aliphatic hydrocarbon polymerization medium. With this aim, a [B(C_6_F_5_)_4_]^−^-based co-catalyst containing long alkyl chains [Me(C_18_H_37_)_2_N-H]^+^[B(C_6_F_5_)_4_]^−^, which is soluble in cyclohexane or methylcyclohexane, was introduced more than two decades ago by Dow [34]. Activated complexes generated with [Me(C_18_H_37_)_2_N-H]^+^[B(C_6_F_5_)_4_]^−^ may be soluble and stable through the coordination of Me(C_18_H_37_)_2_N (a byproduct generated in the activation reaction) with the metal center (i.e., formation of [**L**-M(Me)(NMe(C_18_H_37_)_2_)]^+^[B(C_6_F_5_)_4_]^−^-type species; Scheme 1c) [35,36]. However, [Me(C_18_H_37_)_2_N-H]^+^[B(C_6_F_5_)_4_]^−^ is highly soluble, cannot be purified by recrystallization, and is contaminated with either water or Cl^−^ salt impurities, which interfere with the activation reaction [37]. In this work, we report on a method to prepare high-purity trialkylammonium tetrakis(pentafluorophenyl)borate in an aliphatic hydrocarbon solvent and the activation reactions of some typical metallocene and half-metallocene complexes.

## 2. Results and Discussion

### 2.1. Preparation of [Me(C_18_H_37_)_2_N-H]^+^[B(C_6_F_5_)_4_]^−^

**Scheme 2:**
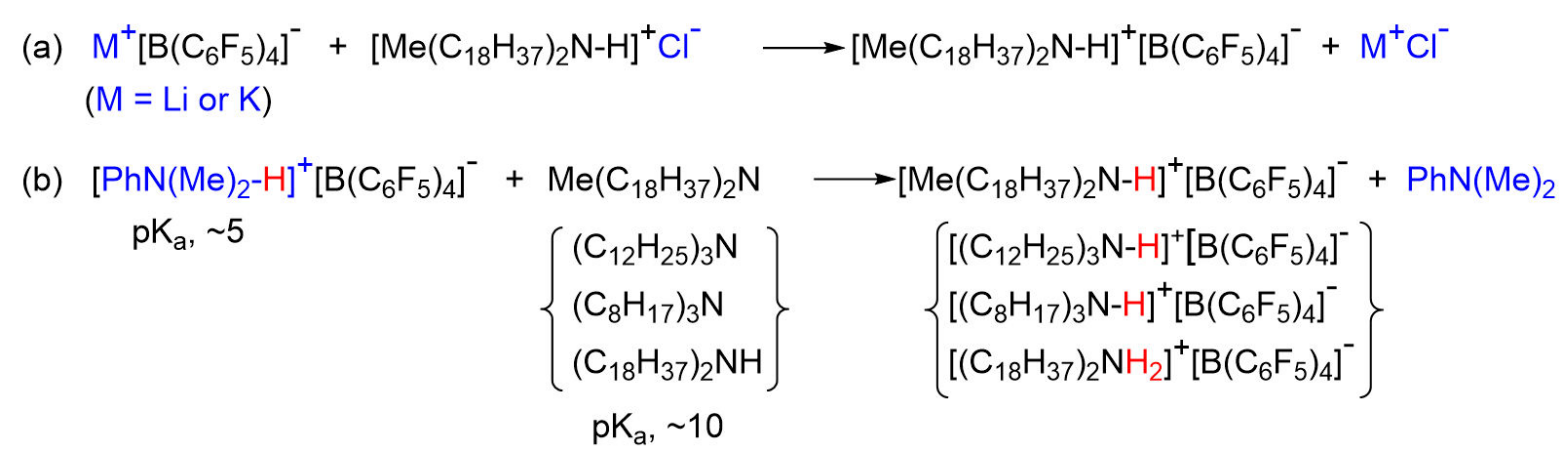
Preparation of [Me(C_18_H_37_)_2_N-H]^+^[B(C_6_F_5_)_4_]^−^ via the conventional salt metathesis reaction (**a**) and via the acid–base reaction disclosed in this work (**b**).

**Figure 1:**
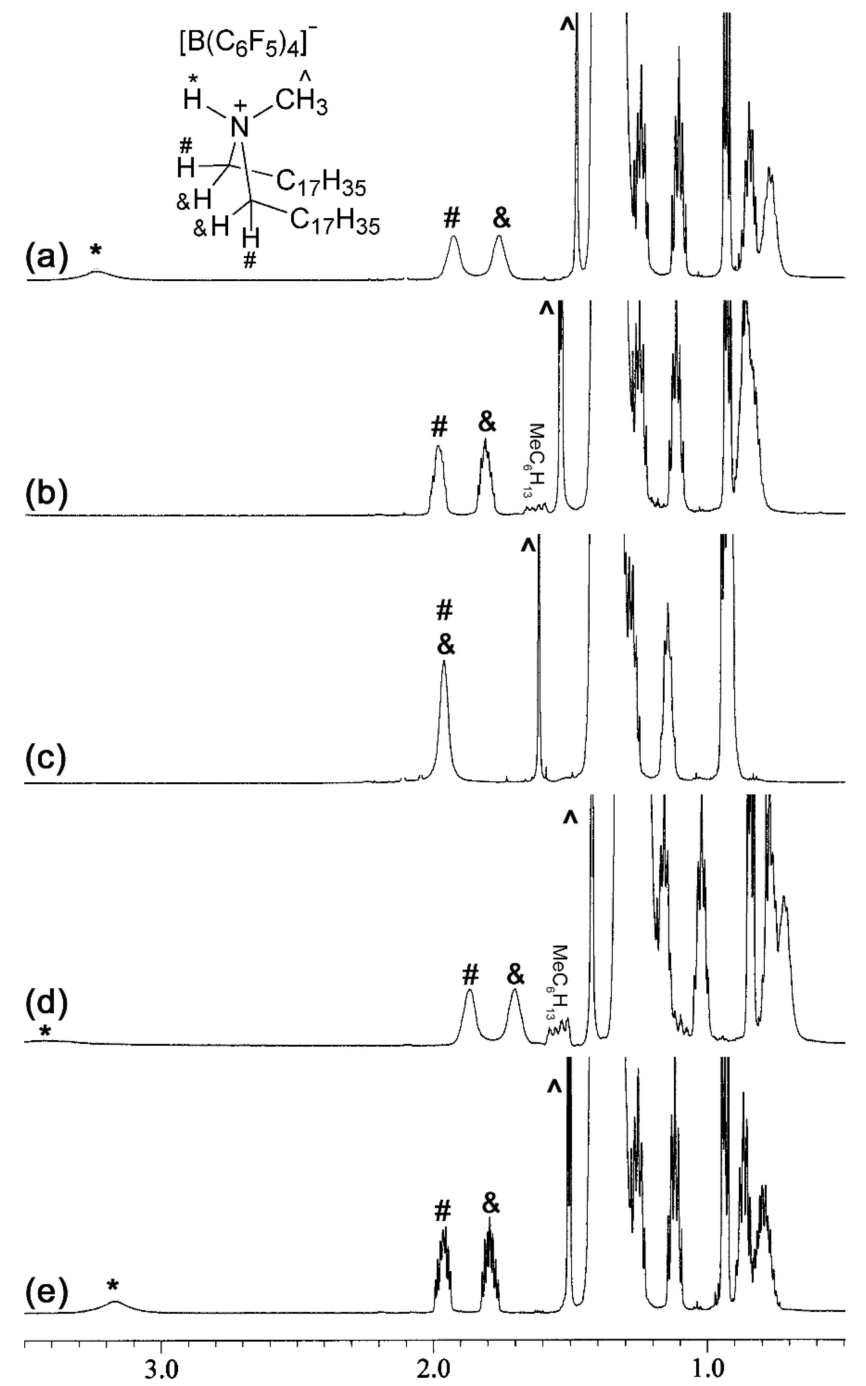
^1^H NMR spectra (recorded in C_6_D_6_) of [Me(C_18_H_37_)_2_N-H]^+^[B(C_6_F_5_)_4_]^−^ prepared by the reactions Li^+^[B(C_6_F_5_)_4_]^−^ + [Me(C_18_H_37_)_2_N-H]^+^[Cl]^−^ (removal of LiCl with water) (**a**), Li^+^[B(C_6_F_5_)_4_]^−^ + [Me(C_18_H_37_)_2_N-H]^+^[Cl]^−^ (removal of LiCl by filtration) (**b**), K^+^[B(C_6_F_5_)_4_]^−^ + [Me(C_18_H_37_)_2_N-H]^+^[Cl]^−^ (removal of KCl with water) (**c**), K^+^[B(C_6_F_5_)_4_]^−^ + [Me(C_18_H_37_)_2_N-H]^+^[Cl]^−^ (removal of KCl by filtration) (**d**), and [PhN(Me)_2_-H]^+^[B(C_6_F_5_)_4_]^−^ + Me(C_18_H_37_)_2_N (**e**). Signals marked with *, #, &, and ^ characters correspond to protons marked with those characters in the structure.

A preparation method for [Me(C_18_H_37_)_2_N-H]^+^[B(C_6_F_5_)_4_]^−^ that uses a salt metathesis reaction between Li^+^[B(C_6_F_5_)_4_]^−^ and [Me(C_18_H_37_)_2_N-H]^+^[Cl]^−^ (Scheme 2a) has been previously reported [34]. In this method, Li^+^[B(C_6_F_5_)_4_]^−^ and [Me(C_18_H_37_)_2_N-H]^+^[Cl]^−^ were reacted in a toluene/water two-phase, and the product, [Me(C_18_H_37_)_2_N-H]^+^[B(C_6_F_5_)_4_]^−^, was isolated by collecting the toluene phase while discarding the water phase containing the byproduct, LiCl. However, the yield was unsatisfactory (71%). Some acidic impurities were concomitantly generated, which were washed out by treatment with Na_2_CO_3_. The NCH_2_ protons were observed as two broad signals at 1.93 and 1.77 ppm in the ^1^H NMR spectrum (Figure 1a); the two geminal protons attached to each α-methylene carbon (i.e., NCH_2_) are diastereotopic. The N-H and NCH_3_ signals were also broad, not showing a splitting pattern, at 3.4–3.1 ppm and 1.5 ppm, respectively. Water impurities might interact with N-H and α-protons on the ammonium unit (NCH_2_ and NCH_3_) through hydrogen bonds, causing broadening of the signals. It was impossible to completely remove water impurities; the NCH_2_ signals were persistently broad even after prolonged evacuation, treatment with desiccants such as molecular sieves, or refluxing toluene solution over the Dean-Stark apparatus. A set of signals assigned to *ortho*-, *para*-, and *meta*-fluorine in the [B(C_6_F_5_)_4_]^−^ ion was clearly observed at −132.22, −161.72, and −165.95 ppm, respectively, in the ^19^F NMR spectrum. No Cl^−^ anions were detected in the product; treatment of [(CH_3_CN)_4_Ag]^+^[B(C_6_F_5_)_4_]^−^ with a diethyl ether solution containing the product did not generate any AgCl precipitates, which indicated that LiCl was thoroughly removed by washing with water (Supplementary Materials Figure S1b).

The byproduct LiCl can also be removed by filtration [38]. The product [Me(C_18_H_37_)_2_N-H]^+^[B(C_6_F_5_)_4_]^−^ is soluble in aliphatic hydrocarbon solvents, such as cyclohexane or methylcyclohexane, while LiCl is insoluble, enabling the removal of LiCl by filtration after performing a metathesis reaction in methylcyclohexane. The yield was satisfactorily high (94%), but the filtration procedure was tedious; the LiCl particles generated were so fine they clogged and even penetrated the Celite pad. Filtration was repeated several times, using a thick Celite pad to obtain a clear solution. In the ^1^H NMR spectrum, the NCH_2_ signals were fairly sharp, showing a splitting pattern, but the N-H signal was absent, possibly due to broadening (Figure 1b). Treatment of [(CH_3_CN)_4_Ag]^+^[B(C_6_F_5_)_4_]^−^ with a solution containing the product changed the clear solution turbid due to the formation of AgCl particles (Supplementary Materials Figure S1c), which indicated that the product was contaminated with Cl^−^ ions.

Attempts to prepare [Me(C_18_H_37_)_2_N-H]^+^[B(C_6_F_5_)_4_]^−^ by using another commercial source of borate salt, K^+^[B(C_6_F_5_)_4_]^−^, gave similar results. In the salt metathesis reaction performed in two water/toluene phases, the yield was low (74%) and the product was contaminated with water, although the Ag^+^ test indicated the absence of Cl^−^ anions (Supplementary Materials Figure S1d). Owing to the presence of water impurities, the two diastereotopic NCH_2_ signals collapsed into a single broad signal (Figure 1c). In the salt metathesis reaction performed in anhydrous methylcyclohexane, the filtration procedure was also tedious, requiring several rounds of filtration to obtain a clear solution, and the product was contaminated with Cl^−^ anions (Supplementary Materials Figure S1e). Two signals were observed for the diastereotopic NCH_2_ protons, but they were broad and did not show any splitting pattern, which was indicative of water contamination in the resulting product (Figure 1d). The commercial source of K^+^[B(C_6_F_5_)_4_]^−^ contained some amount of water impurities, which eventually caused water contamination in the product [Me(C_18_H_37_)_2_N-H]^+^[B(C_6_F_5_)_4_]^−^.

N,N-Dimethylanilinium tetrakis(pentafluorophenyl)borate ([PhN(Me)_2_-H]^+^[B(C_6_F_5_)_4_]^−^) is the chemical most frequently used in commercial processes on a large scale. Although it is prepared via the salt metathesis reaction of M^+^[B(C_6_F_5_)_4_]^−^ (M = Li, K) and [PhN(Me)_2_-H]^+^Cl^−^ [38,39], it can be purified via recrystallization and is available on a large scale as a high-purity crystalline solid. In contrast, [Me(C_18_H_37_)_2_N-H]^+^[B(C_6_F_5_)_4_]^−^ is highly soluble, and there is no purification method. We designed another method to prepare high-purity [Me(C_18_H_37_)_2_N-H]^+^[B(C_6_F_5_)_4_]^−^ from [PhN(Me)_2_-H]^+^[B(C_6_F_5_)_4_]^−^, in which an acid–base reaction was employed instead of the tedious salt metathesis reaction (Scheme 2b). The pK_a_ value of the aniline/anilinium Brønsted base–acid pair is approximately 5, while that of the trialkylammonium/trialkylamine Brønsted acid–base pair is approximately 10, and [PhN(Me)_2_-H]^+^[B(C_6_F_5_)_4_]^−^ could be converted to [Me(C_18_H_37_)_2_N-H]^+^[B(C_6_F_5_)_4_]^−^ by treatment with Me(C_18_H_37_)_2_N in toluene (equilibrium constant anticipated = 10^ΔpKa^, i.e., ~10^5^). The byproduct in this reaction was a neutral compound, PhN(Me)_2_ (boiling point, 194 °C), which could be simply and completely removed by evacuation at 50–60 °C. In this method, it was impossible for the product to be contaminated with Cl^−^ and water once anhydrous reagents and solvents were used. The yield was quantitative, and no Cl^−^ ions were detected in the Ag^+^ test (Supplementary Materials Figure S1a). In the ^1^H NMR spectrum, the NCH_2_ and NCH_3_ signals were observed to be well split (Figure 1e). When water was deliberately added, the well-split NCH_2_ signals were broadened as observed in Figure 1d, inferring that water contamination was a cause of signal broadening (Supplementary Materials Figure S2). Employing the same synthetic protocol, [(C_12_H_25_)_3_N-H]^+^[B(C_6_F_5_)_4_]^−^ and [(C_8_H_17_)_3_N-H]^+^[B(C_6_F_5_)_4_]^−^ were successfully prepared by using (C_12_H_25_)_3_N and (C_8_H_17_)_3_N instead of Me(C_18_H_37_)_2_N (Scheme 2b; Supplementary Materials Figure S3). The former was soluble in methylcyclohexane, whereas the latter was not. The secondary amine-derived [(C_18_H_37_)_2_NH_2_]^+^[B(C_6_F_5_)_4_]^−^, which is soluble in methylcyclohexane, was also obtained by using the same synthetic protocol (Supplementary Materials Figure S4), whereas primary amines such as (C_17_H_35_)_2_C(H)NH_2_ were not effective in the synthetic protocol.

### 2.2. Preparation of L-M(Me)_2_ and L-MCl(Me)-Type Complexes for Activation Studies

**Scheme 3:**
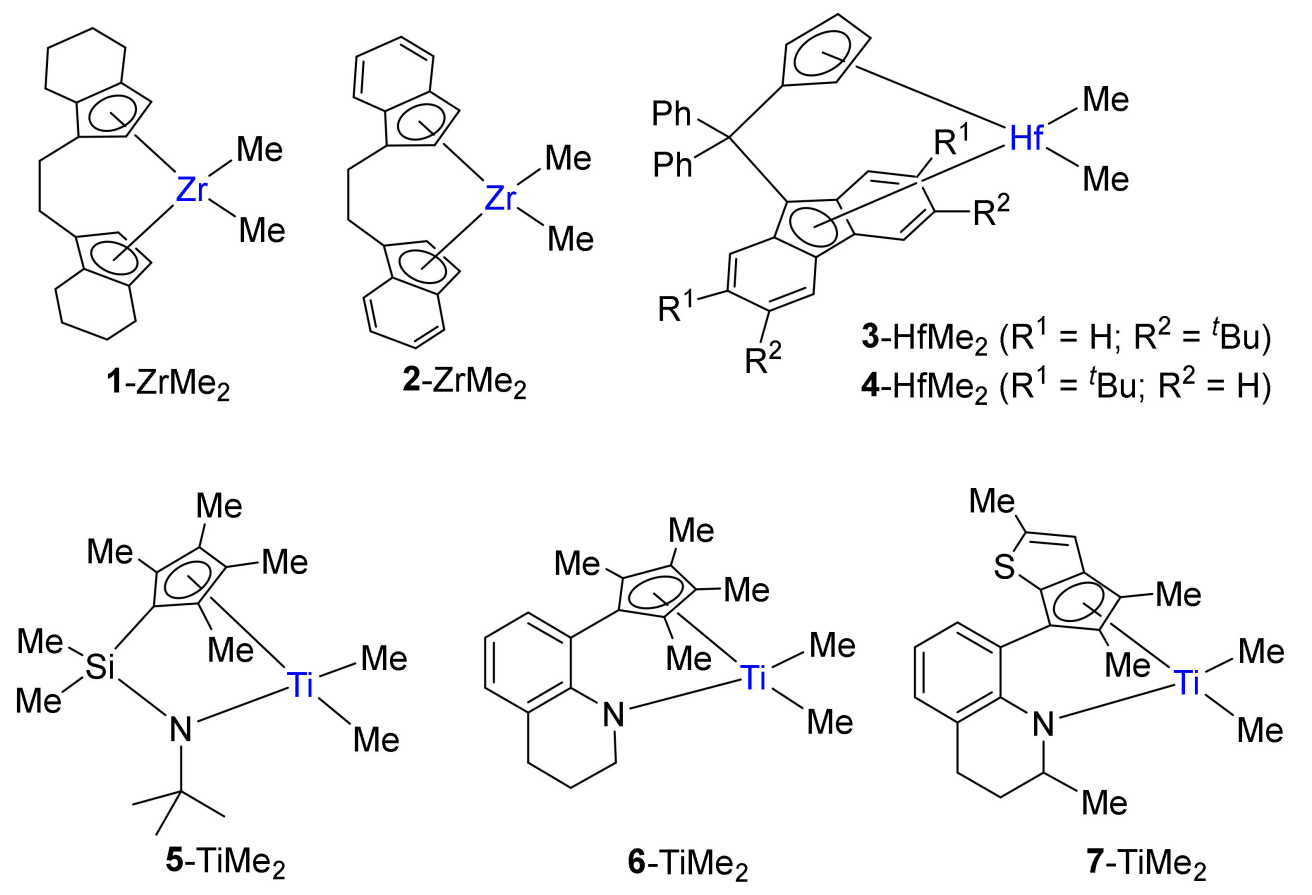
Metallocene and half-metallocene complexes studied in the activation and polymerization reactions.

**Figure 2:**
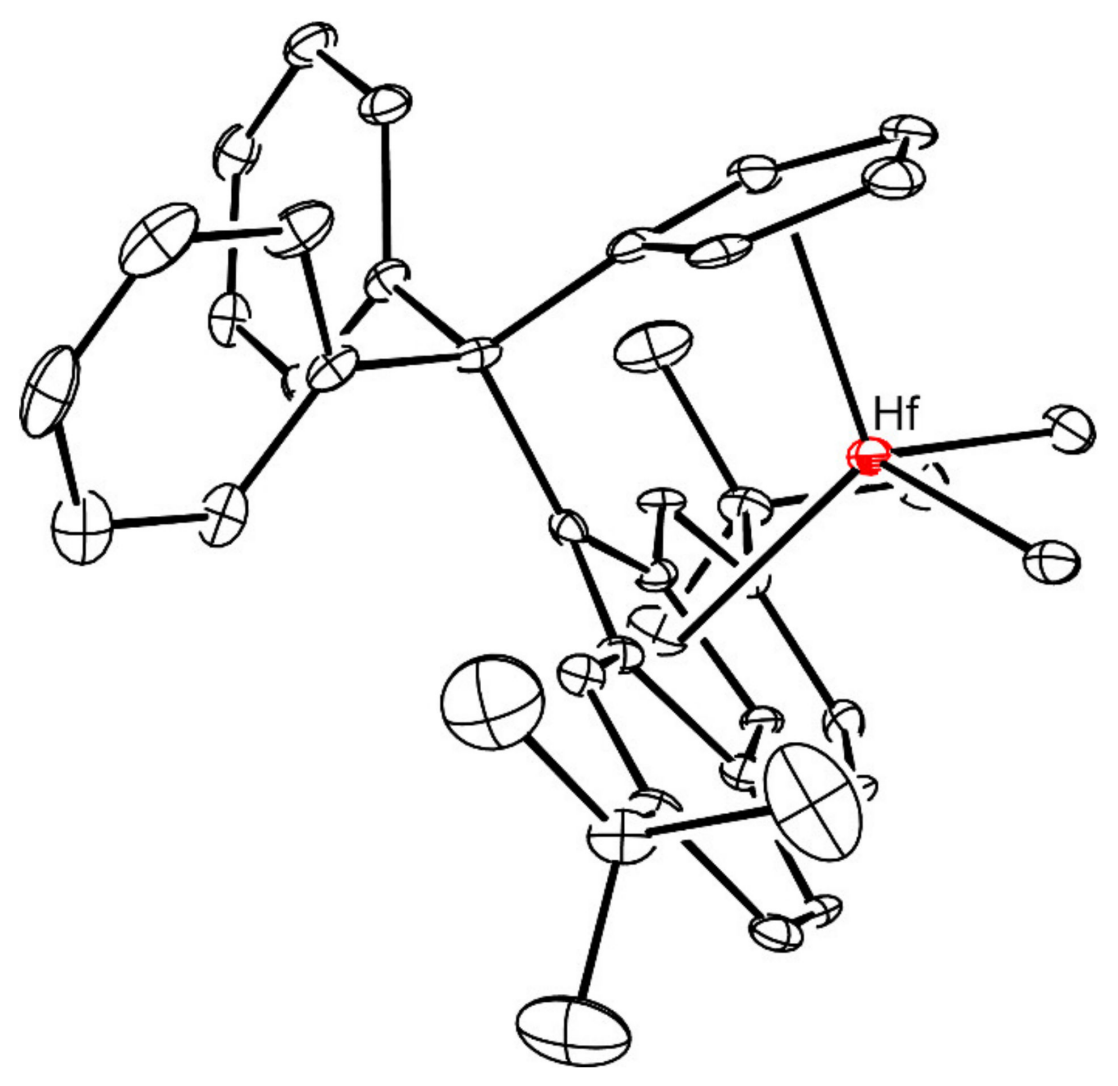
Structure of **4**-HfMe_2_ revealed by X-ray crystallography.

In most cases in industry, as well as in the laboratory, olefin polymerizations have been performed with in situ–generated activated species with neither isolation of the activated species nor monitoring of the activation reaction, because the activated species (i.e., ion pair complexes) are, in most cases, unstable or insoluble in hydrocarbon solvents. In this work, NMR spectroscopy was used to monitor the activation reactions of commercial process-relevant metallocene and half-metallocene complexes (Scheme 3) with the prepared high-purity [Me(C_18_H_37_)_2_N-H]^+^[B(C_6_F_5_)_4_]^−^, which did not contain any water or Cl^−^ impurities. Complexes *rac*-[ethylenebis(tetrahydroindenyl)]Zr(Me)_2_ (**1**-ZrMe_2_) and *rac*-[ethylenebis(indenyl)]Zr(Me)_2_ (**2**-ZrMe_2_) are a representative among metallocene complexes. Hafnocene complexes [Ph_2_C(Cp)(3,6-*^t^*Bu_2_Flu)]Hf(Me)_2_ (**3**-HfMe_2_) and [Ph_2_C(Cp)(2,7-*^t^*Bu_2_Flu)]Hf(Me)_2_ (**4**-HfMe_2_) were prepared in this work. **L**-MCl_2_-type complexes are the most common source of olefin polymerization catalyst precursors, which have been conventionally converted to **L**-M(Me)_2_-type complexes by the action of MeLi or MeMgCl. Although **3**-HfMe_2_ and **4**-HfMe_2_ could be prepared via the conventional method (i.e., reacting **L**-MCl_2_-type complexes with MeMgCl), they were prepared in a more facile and direct manner from ligand precursors **3**-Li_2_ and **4**-Li_2_ in one pot; they gave a moderate yield (41%) when **3**-Li_2_ and **4**-Li_2_ were treated with HfCl_4_ in the presence of two equivalents of MeMgBr [40]. The structure of **4**-HfMe_2_ was confirmed by X-ray crystallography, as shown in Figure 2.

**Scheme 4:**
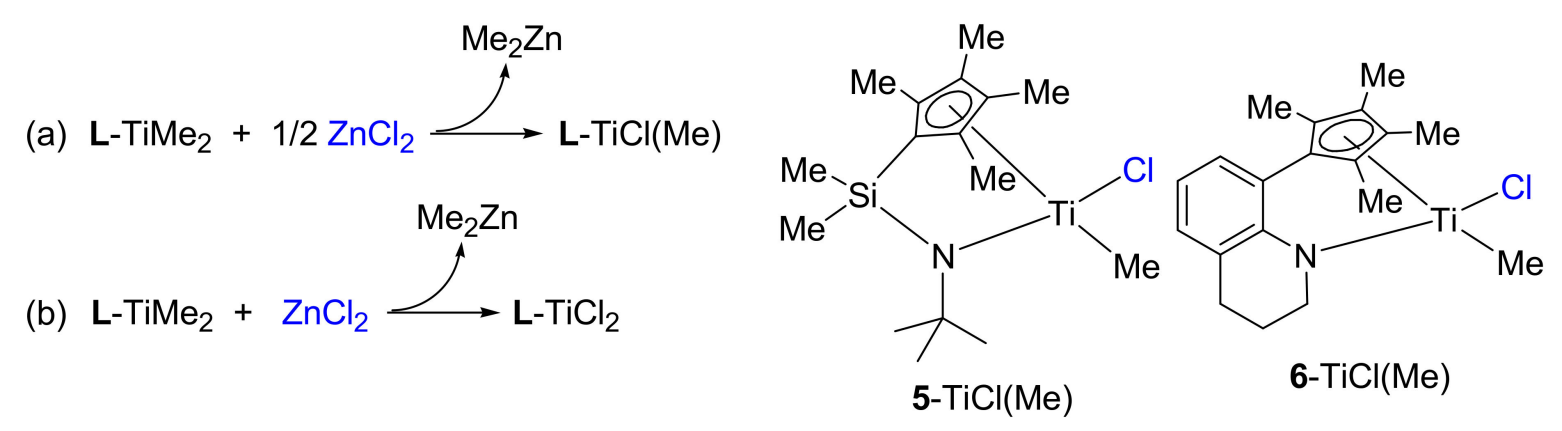
Preparation of L-TiCl(Me) (**a**) and L-TiCl_2_ (**b**) types half-metallocene complexes.

**Figure 3:**
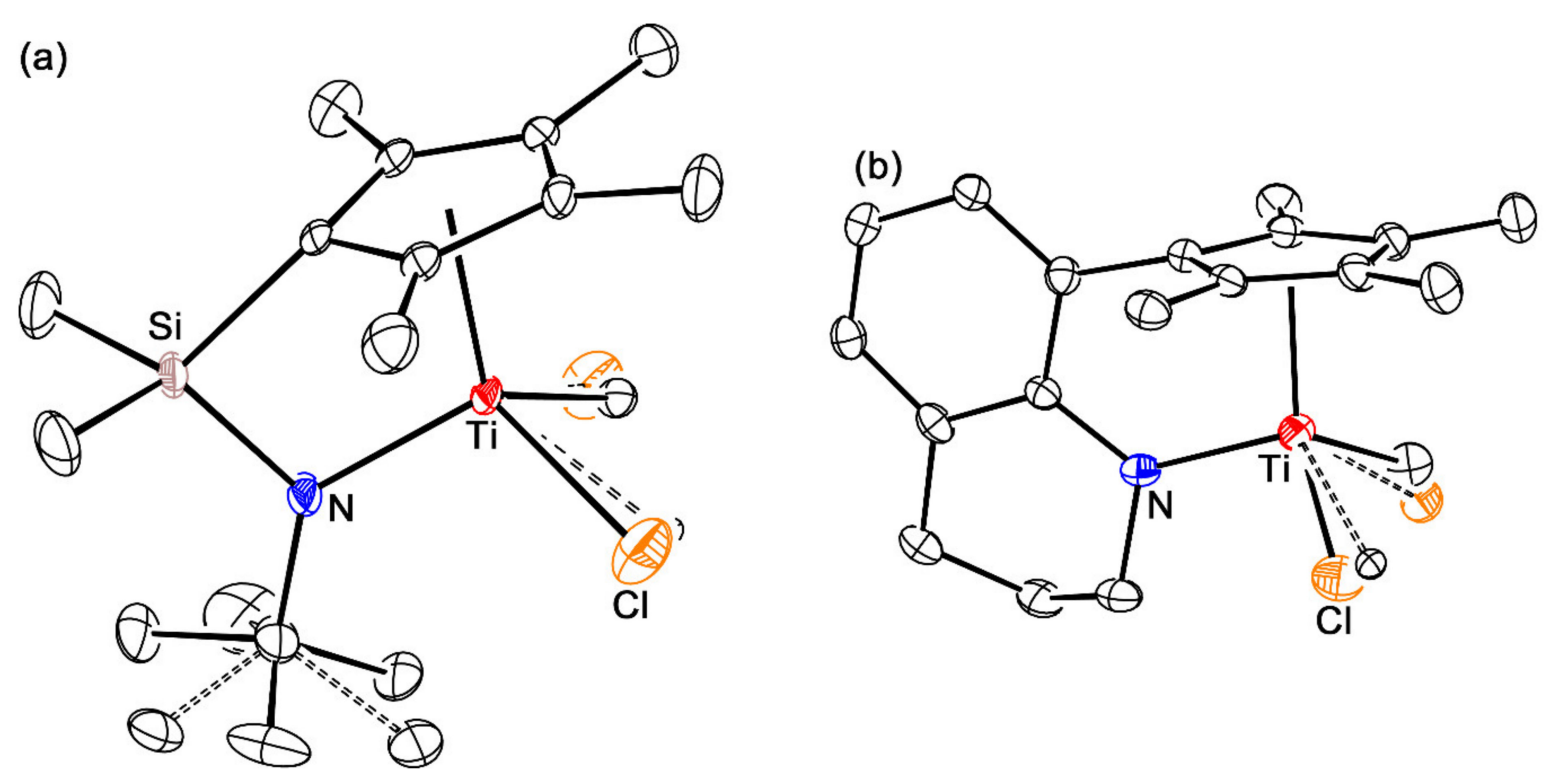
Structures of **5**-TiCl(Me) (**a**) and **6**-TiCl(Me) (**b**) revealed by X-ray crystallography.

Complex [(η^5^-Me_4_C_5_)Si(Me)_2_(κ-N^t^Bu)]Ti(Me)_2_ (**5**-TiMe_2_) is a representative among half-metallocene complexes, named CGC (Constrained Geometry Complex), and tetrahydroquinoline-derived half-metallocene complex [(η^5^-Me_4_C_5_)(C_9_H_9_(κ-N))]Ti(Me)_2_ (**6**-TiMe_2_) and thiophene-fused cyclopentadienyl Ti complex [(η^5^-Me_3_C_7_H_1_S)(C_10_H_11_(κ-N))]Ti(Me)_2_ (**7**-TiMe_2_) are the ones developed in our laboratory. Half-metallocene complexes **5**-TiMe_2_, **6**-TiMe_2_, and **7**-TiMe_2_ were also obtained in good yields when **L**-Li_2_ or **L**-(MgCl)_2_ were treated with TiCl_4_ in the presence of two equivalents of MeMgCl [41,42,43,44]. It was attempted to prepare **L**-MCl(Me)-type complexes. When **6**-(MgCl)_2_ was treated with TiCl_4_(DME) in the presence of one equivalent of MeMgCl with the aim of obtaining **6**-TiCl(Me), the desired complex was generated but contaminated with a significant amount of **6**-TiMe_2_ (1:2.5). The desired **6**-TiCl(Me) complex was cleanly generated when **6**-TiMe_2_ was treated with a half equivalent ZnCl_2_ in toluene or C_6_D_6_ (Scheme 4a; Supplementary Materials Figure S5). The byproduct Me_2_Zn signal was observed at −0.68 ppm, which could be removed during the solvent removal step (boiling point of Me_2_Zn, 46 °C). Complex **6**-TiCl(Me) was stable in C_6_D_12_, that is, the disproportionation of **6**-TiCl(Me) to **6**-TiMe_2_ and **6**-TiCl_2_ species did not occur in C_6_D_12_. Complex **5**-TiCl(Me) was also prepared by using the same method. ^1^H and ^13^C NMR spectra agreed with the structure (Supplementary Materials Figure S5), and the structures of **5**-TiCl(Me) and **6**-TiCl(Me) were confirmed by X-ray crystallography, although the TiCl(Me) fragments were disordered (Figure 3). **L**-TiCl_2_-type half-metallocene complexes (e.g., **5**-TiCl_2_ and **6**-TiCl_2_) are not yielded when **L**-Li_2_ or **L**-(MgCl)_2_ was reacted directly with TiCl_4_, although the corresponding reaction in the presence of two equivalents of MeMgCl afforded the **L**-TiMe_2_-type complexes in good yields. Reported preparation method for **5**-TiCl_2_ was a tedious and costly two-step process: reacting **5**-Li_2_ with TiCl_3_(THF)_2_ to obtain **5**-Ti^III^Cl, which was transformed to **5**-TiCl_2_ by treatment with PbCl_2_ or AgCl [45]. Preparation of **6**-TiCl_2_ was not reported yet. In this work, it was also demonstrated that **5**-TiMe_2_ and **6**-TiMe_2_ could be easily transformed to **5**-TiCl_2_ and **6**-TiCl_2_, respectively, through the treatment of an equimolar amount of ZnCl_2_ (Scheme 4b; Supplementary Materials Figure S6).

### 2.3. Activation Reactions

**Figure 4:**
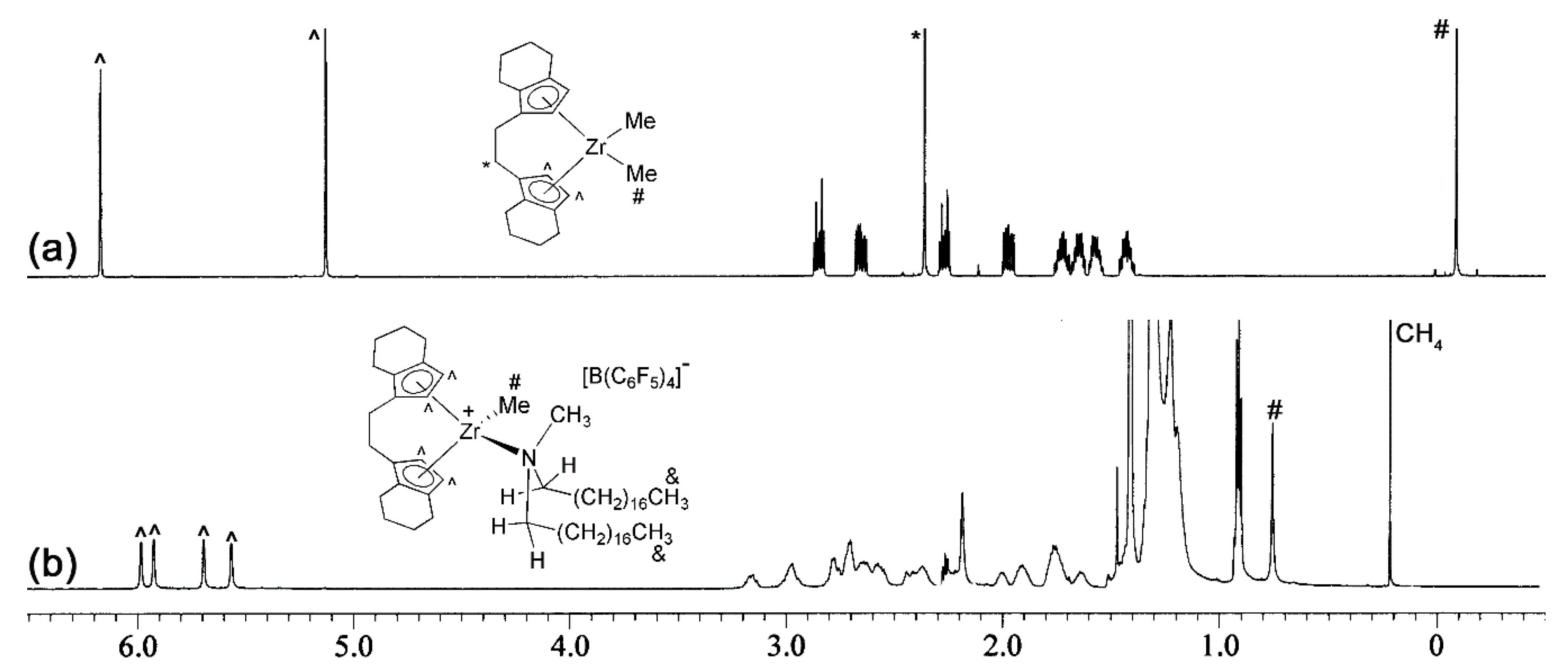
^1^H NMR spectra of **1**-ZrMe_2_ (**a**) and its activated complex [**1**-Zr(Me)(N(Me)(C_18_H_37_)_2_)]^+^[B(C_6_F_5_)_4_]^−^ (**b**). Signals marked with ^, *, and # characters correspond to protons marked with those characters in the structure.

In the activation reaction of **1**-ZrMe_2_ with [Me(C_18_H_37_)_2_N-H]^+^[B(C_6_F_5_)_4_]^−^, performed with the aromatic hydrocarbon solvent C_6_D_6_ in a sealed NMR tube, the ^1^H NMR spectral signals assigned to **1**-ZrMe_2_ completely disappeared with the generation of methane, but the generated signals were too complicated to be assigned to a single species. However, when the reaction was performed in the aliphatic hydrocarbon solvent C_6_D_12_, a set of signals that could be assigned to the desired activated ion pair complex, [**1**-Zr(Me)(NMe(C_18_H_37_)_2_)]^+^[B(C_6_F_5_)_4_]^−^, was cleanly generated with a methane signal (Figure 4). The byproduct amine Me(C_18_H_37_)_2_N might loosely coordinate to the cationic Zr center, making the signals relatively broad. By coordination with Me(C_18_H_37_)_2_N, the two indenyl moieties are inequivalent, leading to the observation of four indenyl protons and carbons separately at 5.97, 5.91, 5.68, and 5.55 ppm as a broad singlet in the ^1^H NMR spectrum (Figure 4b and Supplementary Materials Figure S7) and 115.8, 114.8, 113.0, and 109.2 ppm in ^13^C NMR spectrum (Supplementary Materials Figures S8 and S9). Signal for Zr-CH_3_ was observed at 0.73 ppm as a singlet in the ^1^H NMR spectrum and at 45.7 ppm in the ^13^C NMR spectrum. In the ^19^F NMR spectrum, a set of clean signals assignable to *ortho*-, *para*-, and *meta*-fluorine of -C_6_F_5_ were observed at −132.0, −163.6, and −167.2 ppm (Supplementary Materials Figure S10), indicating that the [B(C_6_F_5_)_4_]^−^ anion was not destroyed during the activation reaction. The activated complex was stable in C_6_D_12_; the signals assigned to [**1**-Zr(Me)(N(Me)(C_18_H_37_)_2_)]^+^[B(C_6_F_5_)_4_]^−^ were persistently observed with no generation of other signals, even after a week. In contrast, many unidentified solids were deposited in the reaction of **2**-ZrMe_2_. In the C_6_D_12_ solution phase, the signals that were assigned to the desired [**2**-Zr(Me)(NMe(C_18_H_37_)_2_)]^+^[B(C_6_F_5_)_4_]^−^ were observed at the initial stage, but disappeared overnight along with the complicated signals that could not be interpreted.

**Figure 5:**
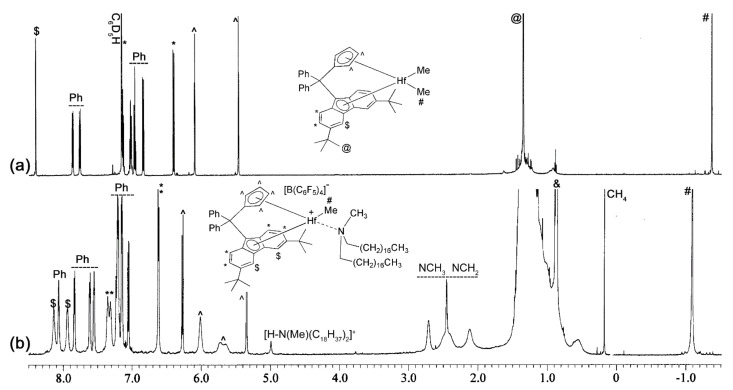
^1^H NMR spectra of **3**-HfMe_2_ (**a**) and its activated complex [**3**-Hf(Me)(NMe(C_18_H_37_)_2_)]^+^[B(C_6_F_5_)_4_]^−^ (**b**). Signals marked with $, *, ^, @, &, and # characters correspond to protons marked with those characters in the structure.

When **3**-HfMe_2_ was reacted with [Me(C_18_H_37_)_2_N-H]^+^[B(C_6_F_5_)_4_]^−^ in C_6_D_12_, analysis of the ^1^H NMR spectrum indicated that the desired ion-pair complex [**3**-Hf(Me)(NMe(C_18_H_37_)_2_)]^+^[B(C_6_F_5_)_4_]^−^ was cleanly generated (Figure 5). Owing to the coordination of Me(C_18_H_37_)_2_N with a vacant site of the Hf center, four cyclopentadienyl protons and six fluorenyl protons were independently observed. Signals corresponding to NCH_3_ and NCH_2_ were very broad at 1.89–2.93 ppm, indicating that Me(C_18_H_37_)_2_N loosely interacts with the Hf center; Me(C_18_H_37_)_2_N might be under fluxional between a coordinated state and an uncoordinated state while occurring the site epimerization. For free Me(C_18_H_37_)_2_N, signals corresponding to NCH_3_ and NCH_2_ were observed in the same region (2.10 and 2.23 ppm) as singlet and triplet, respectively (Supplementary Materials Figure S11). The activated complex was stable as a solution in C_6_D_12_; the ^1^H NMR spectrum was unaltered when recorded after several weeks. Analysis of the ^1^H NMR spectrum indicated that the desired ion-pair complex [**4**-Hf(Me)(NMe(C_18_H_37_)_2_)]^+^[B(C_6_F_5_)_4_]^−^ was also generated in the reaction of **4**-HfMe_2_ and [Me(C_18_H_37_)_2_N-H]^+^[B(C_6_F_5_)_4_]^−^ (Supplementary Materials Figure S12). However, in this case, some insoluble fractions were concomitantly generated. We suspected that the insoluble fraction was a dinuclear adduct of [**4**-Hf(Me)]^+^[B(C_6_F_5_)_4_]^−^ and **4**-HfMe_2_ (i.e., [**4**-Hf(Me)(μ-Me)(**4**-Hf(Me))]^+^[B(C_6_F_5_)_4_]^−^, which, however, disappeared after 1 d, becoming soluble when 1.5 equiv [Me(C_18_H_37_)_2_N-H]^+^[B(C_6_F_5_)_4_]^−^ was added to the activation reaction. In the ^1^H NMR spectra, a signal at ~5.1 ppm which corresponded to N-H in the remaining [Me(C_18_H_37_)_2_N-H]^+^[B(C_6_F_5_)_4_]^−^ was persistently observed, indicating further reaction of the activated species [**3**- and **4**-Hf(Me)(NMe(C_18_H_37_)_2_)]^+^[B(C_6_F_5_)_4_]^−^ with [Me(C_18_H_37_)_2_N-H]^+^[B(C_6_F_5_)_4_]^−^ was not allowed (Figure 5 and Supplementary Materials Figure S12). The N-H proton in [Me(C_18_H_37_)_2_N-H]^+^[B(C_6_F_5_)_4_]^−^ was observed as a broad signal at ~3.2 ppm in C_6_D_6_ (Figure 1e) which, however, was shifted to ~5.1 ppm when recorded in C_6_D_12_ (Supplementary Materials Figure S11).

**Scheme 5:**
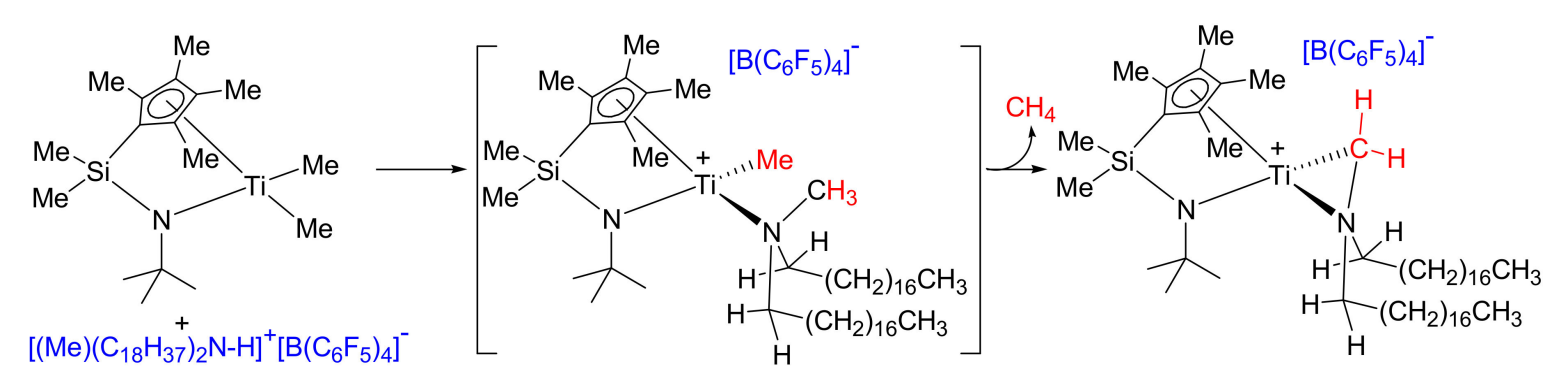
Activation reaction of a half-metallocene complex.

The activation reaction of half-metallocene titanium complexes is complicated. When **5**-TiMe_2_ was reacted with an equivalent [Me(C_18_H_37_)_2_N-H]^+^[B(C_6_F_5_)_4_]^−^ in C_6_D_12_, the reactant signals in the ^1^H NMR spectrum immediately disappeared with the generation of the CH_4_ signal, but the signals were too complicated to be interpreted. However, they converged to a set of assignable signals overnight (Supplementary Materials Figure S13). The generated species was not the desired ion-pair complex [**5**-Ti(Me)(NMe(C_18_H_37_)_2_)]^+^[B(C_6_F_5_)_4_]^−^; the Ti-CH_3_ signal was not observed, but, instead, two broad signals were observed at 0.45 and −0.71 ppm, whose intensity was 1/3 relative to that of the C_5_CH_3_ or SiCH_3_ signal. We tentatively assigned the generated species to [**5**-Ti(CH_2_N(C_18_H_37_)_2_)]^+^[B(C_6_F_5_)_4_]^−^, which may have been formed from [**5**-Ti(Me)(NMe(C_18_H_37_)_2_)]^+^[B(C_6_F_5_)_4_]^−^ via an intramolecular σ-bond metathesis reaction with the generation of CH_4_ (Scheme 5). With the species [**5**-Ti(CH_2_N(C_18_H_37_)_2_)]^+^[B(C_6_F_5_)_4_]^−^, the four NCH_2_ protons, the four methyl groups attached to cyclopentadienyl ligand, and the two methyl groups attached to Si are separately observed at 3.29, 2.93, 2.77, 2.51, 2.20, 2.08, 1.88, 1.83, 0.75, and 0.58 ppm with intensity ratios of roughly 1:1:1:1:3:3:3:3:3:3, respectively, along with Ti-CH_2_ signals at 0.45 and −0.71 ppm with intensity ratios of roughly 1 and 1, respectively. The same type of complex, [Cp_2_Zr(CH_2_N(C_18_H_37_)_2_)]^+^[B(C_6_F_5_)_4_]^−^, was reported for the activation reaction of Cp_2_ZrMe_2_ [46,47,48], and a similar transformation of [**L**-M(Me)]^+^[B(C_6_F_5_)_4_]^−^-type complexes to other species (via the σ-bond metathesis reaction with formation of other M-C bonds and generation of CH_4_) has also been reported for other metallocene and post-metallocene complexes [31,49,50,51]. Action of [Me(C_18_H_37_)_2_N-H]^+^[B(C_6_F_5_)_4_]^−^ to Hf-based metallocene (e.g., **3**- and **4**-HfMe_2_), half-metallocene (e.g., **6**-HfMe_2_), and post-metallocene (e.g., pyridylamido-HfMe_2_) complexes usually afforded stable [**L**-Hf(Me)(NMe(C_18_H_37_)_2_)]^+^[B(C_6_F_5_)_4_]^−^ type ion pair complexes [37]. In contrast, stability of [**L**-Zr(Me)(NMe(C_18_H_37_)_2_)]^+^[B(C_6_F_5_)_4_]^−^ type ion pair complexes generated from Zr-based metallocenes (e.g., **1**-ZrMe_2_, **2**-ZrMe_2_, Cp_2_ZrMe_2_) depended on the ligand structure while those species generated from Ti-based half-metallocene complexes were unstable in all cases (see also below). These stability difference might be attributed to M-C bond strength, which increases by moving from Ti to Zr and further to Hf.

**Figure 6:**
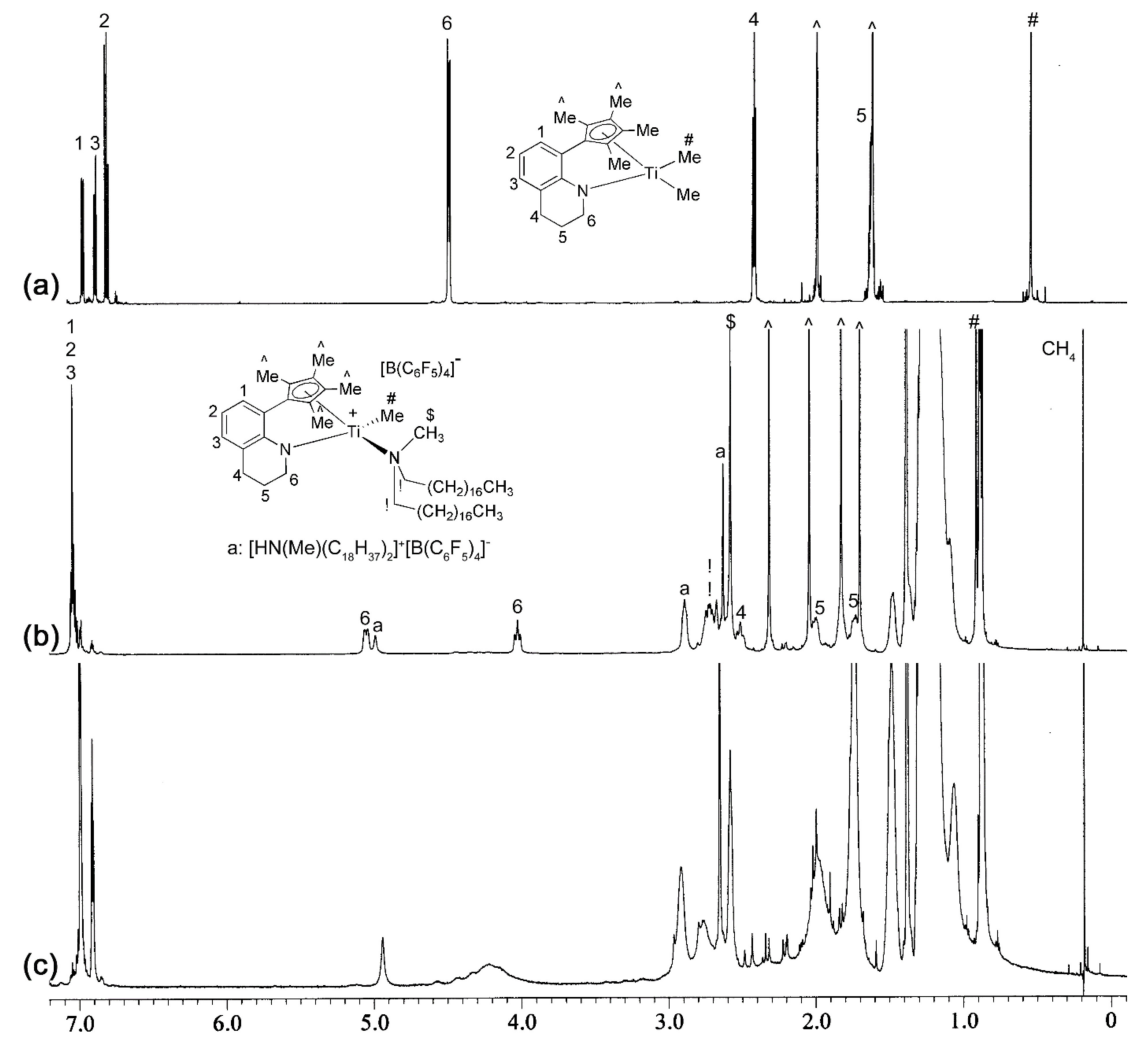
^1^H NMR spectra of **6**-TiMe_2_ (**a**) and its activated complex [**6**-Ti(CH_2_N(C_18_H_37_)_2_)]^+^[B(C_6_F_5_)_4_]^−^ formed at an initial stage (**b**) and transformed to another species overnight (**c**). Signals marked with !, $, ^, and # characters correspond to protons marked with those characters in the structure.

The addition of 1.5 equiv [Me(C_18_H_37_)_2_N-H]^+^[B(C_6_F_5_)_4_]^−^ to **6**-TiMe_2_ afforded a clean set of signals at an initial stage (<15 min) that were assigned to the desired activated complex [**6**-Ti(Me)(NMe(C_18_H_37_)_2_)]^+^[B(C_6_F_5_)_4_]^−^ (Figure 6b) [37]. The tetrahydroquinoline-NCH_2_ protons were separately observed at 5.04 and 4.01 ppm and four methyl groups attached to cyclopentadienyl ligand were observed independently at 2.31, 2.04, 1.82, and 1.70 ppm. The signal corresponding to Ti-CH_3_ was observed at 0.91 ppm. However, overnight, the signals corresponding to [**6**-Ti(Me)(NMe(C_18_H_37_)_2_)]^+^[B(C_6_F_5_)_4_]^−^ disappeared completely and were converted to a new set of signals that were too broad to be interpreted(Figure 6c). We hypothesized that [**6**-Ti(Me)(NMe(C_18_H_37_)_2_)]^+^[B(C_6_F_5_)_4_]^−^ was converted to [**6**-Ti(CH_2_N(C_18_H_37_)_2_)]^+^[B(C_6_F_5_)_4_]^−^ overnight as in the activation reaction of **5**-TiMe_2_. An activation reaction with 1 equiv [Me(C_18_H_37_)_2_N-H]^+^[B(C_6_F_5_)_4_]^−^ instead of 1.5 equiv afforded similar results, with no generation of an insoluble fraction, but several other tetrahydroquinoline-NCH_2_ signals were observed in the 3.54–4.76 ppm region, along with the main ones assigned to [**6**-Ti(Me)(NMe(C_18_H_37_)_2_)]^+^[B(C_6_F_5_)_4_]^−^. The reaction of **6**-TiMe_2_ with the bulkier [(C_12_H_25_)_3_N-H]^+^[B(C_6_F_5_)_4_]^−^ was not clean; many insoluble species were deposited in C_6_D_12_ and the ^1^H NMR spectrum signals were too complicated to be interpreted. In the reaction pot of **6**-TiMe_2_ with secondary amine-derived salt [(C_18_H_37_)_2_NH_2_]^+^[B(C_6_F_5_)_4_]^−^ in C_6_D_12_, two species were observed at the initial stage with no generation of insoluble fractions, which were assigned to [**6**-Ti(Me)(N(H)(C_18_H_37_)_2_)]^+^[B(C_6_F_5_)_4_]^−^ and [**6**-Ti(N(C_18_H_37_)_2_)]^+^[B(C_6_F_5_)_4_]^−^ (Supplementary Materials Figure S14). However, after 2 h, the signals assigned to the former completely disappeared, cleanly leaving a set of signals assigned to the latter. The reaction pattern between **7**-TiMe_2_ and [Me(C_18_H_37_)_2_N-H]^+^[B(C_6_F_5_)_4_]^−^ was similar to that of **6**-TiMe_2_ (Supplementary Materials Figure S15).

**Figure 7:**
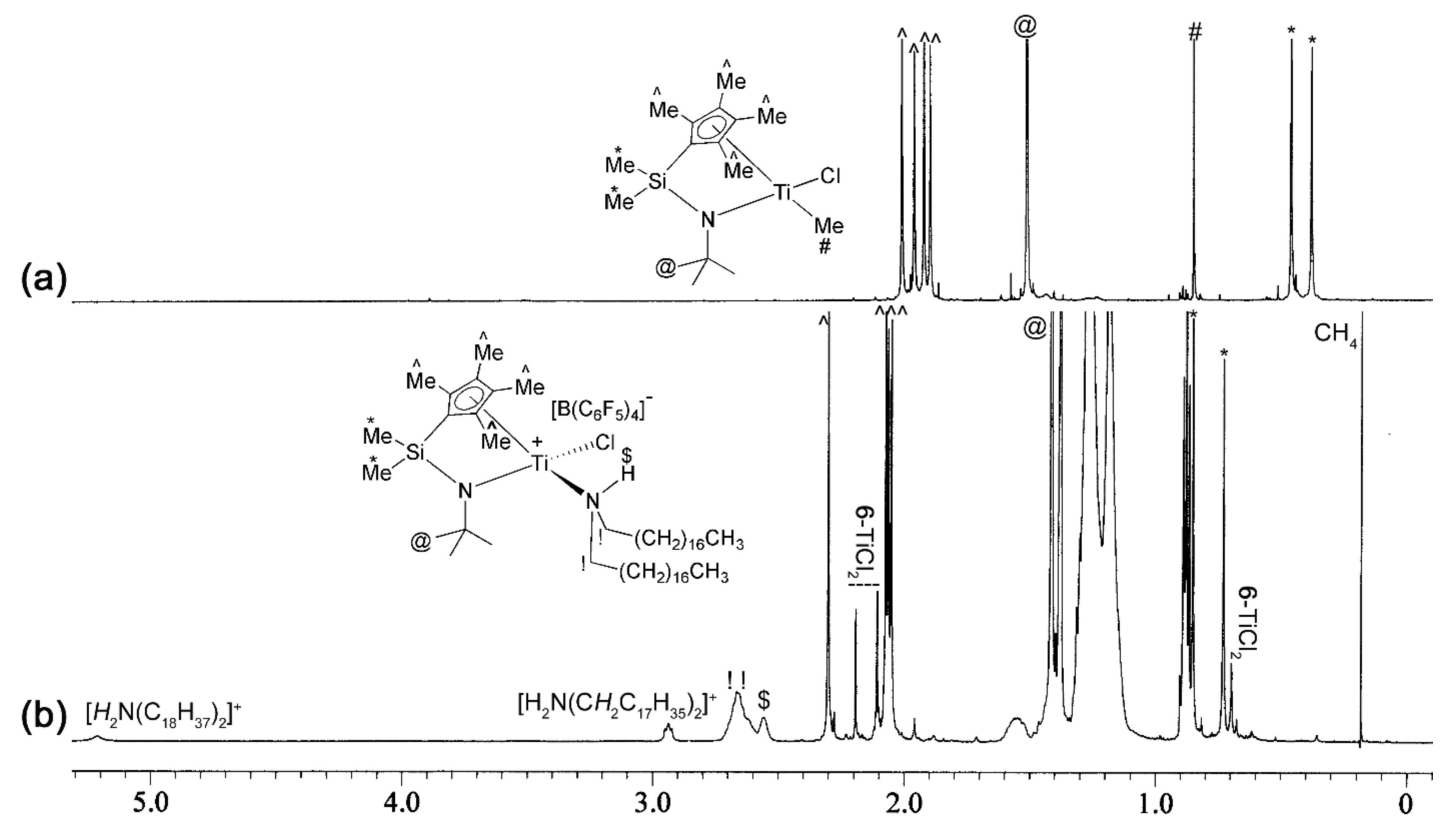
^1^H NMR spectra of **5**-TiCl(Me) (**a**) and its activated complex [**5**-TiCl(N(H)(C_18_H_37_)_2_)]^+^[B(C_6_F_5_)_4_]^−^ (**b**). Signals marked with !, $, ^, @, #, and * characters correspond to protons marked with those characters in the structure.

For **L**-TiMe_2_-type half-metallocene complexes, the desired activated complexes, [**L**-Ti(Me)(NMe(C_18_H_37_)_2_)]^+^[B(C_6_F_5_)_4_]^−^, were unstable and slowly transformed to other species that we tentatively assigned to [**L**-Ti(CH_2_N(C_18_H_37_)_2_)]^+^[B(C_6_F_5_)_4_]^−^. To prevent further reactions with [**L**-Ti(Me)(NMe(C_18_H_37_)_2_)]^+^[B(C_6_F_5_)_4_]^−^, **L**-MCl(Me)-type complexes were prepared and reacted with [Me(C_18_H_37_)_2_N-H]^+^[B(C_6_F_5_)_4_]^−^. The reaction of **6**-TiCl(Me) with [Me(C_18_H_37_)_2_N-H]^+^[B(C_6_F_5_)_4_]^−^ in C_6_D_12_ did not afford a single species, many insoluble fractions were generated, and the ^1^H NMR signals were too broad and too complicated to be interpreted. In contrast, the reaction of **6**-TiCl(Me) with secondary amine-derived [(C_18_H_37_)_2_NH_2_]^+^[B(C_6_F_5_)_4_]^−^ (1.1 equiv) afforded a clean set of signals, which could be assigned to the desired complex [**6**-TiCl(N(H)(C_18_H_37_)_2_)]^+^[B(C_6_F_5_)_4_]^−^, with no generation of insoluble fractions (Supplementary Materials Figure S5); the signal pattern was similar to that observed for [**6**-Ti(Me)(NMe(C_18_H_37_)_2_)]^+^[B(C_6_F_5_)_4_]^−^ (Figure 6b) with tetrahydroquinoline-NCH_2_ signals observed at 5.04 and 4.01 ppm and the TiCH_3_ signal absent. Signals assigned to **6**-TiCl_2_ were also observed at approximately 10 mol%. The addition of 1.1 equiv [(C_18_H_37_)_2_NH_2_]^+^[B(C_6_F_5_)_4_]^−^ to **5**-TiCl(Me) also resulted in the generation of the desired complex [**5**-TiCl(N(H)(C_18_H_37_)_2_)]^+^[B(C_6_F_5_)_4_]^−^ (Figure 7); a signal corresponding to Ti-CH_3_ in the reactant disappeared completely and the CH_4_ signal appeared along with a shift of C_5_CH_3_ signals. NH and NCH_2_ signals were observed at 2.75–2.51. A set of signals that could be assigned to **5**-TiCl_2_ was also observed at an amount of approximately 10 mol%, as was observed in the activation reaction of **6**-TiCl(Me).

### 2.4. Polymerization Studies

The performance of the activated species was tested in an olefin polymerization performed in a small-sized (75 mL) bomb reactor under 20 bar of ethylene gas by using hexane solvent (15.5 g) and 1-octene comonomer (5.0 g) with a small amount of trioctylaluminum as a scavenger (Al/**L**-M(Me)_2_ = 33). The temperature could not be controlled; it increased from 65 °C up to 164 °C within several minutes due to the heat generated by the exothermic reaction (Table 1). Instead, temperature was recorded in an isothermal 110 °C bath, which enabled us to qualitatively monitor the performance of the catalyst. When [**1**-Zr(Me)(NMe(C_18_H_37_)_2_)]^+^[B(C_6_F_5_)_4_]^−^ (1.0 μmol) was fed into the reactor at a temperature of 65 °C, the temperature increased immediately, reaching 116 °C in 2 min, and then gradually decreased, reaching 94 °C after 15 min, when the polymerization was quenched. A large amount of polymer was formed as a form of slurry (9.3 g; entry 1), which indicated that 1-octene incorporation was not significant. In contrast, when [**3**-Hf(Me)(NMe(C_18_H_37_)_2_)]^+^[B(C_6_F_5_)_4_]^−^ was fed, the temperature slowly increased for 10 min until reaching 82 °C, after which, the temperature increased dramatically, reaching 164 °C in 2 min, after which the temperature gradually decreased to 91 °C during the rest 13 min polymerization time (entry 2). Amine NMe(C_18_H_37_)_2_ should be detached from the Hf center to initiate polymerization, which might require a temperature of at least 80 °C. Even with the much higher temperature rise (164 vs. 116 °C) relative to [**1**-Zr(Me)(NMe(C_18_H_37_)_2_)]^+^[B(C_6_F_5_)_4_]^−^, the amount of generated polymer was less (5.2 g vs. 9.3 g). In the case of [**3**-Hf(Me)(NMe(C_18_H_37_)_2_)]^+^[B(C_6_F_5_)_4_]^−^, the generated polymer was not a slurry but a form dissolved in hexane, indicating that 1-octene incorporation was significant. In fact, the 1-octene content (*F*_C8_) in the generated copolymer was significantly higher than that in the copolymer generated with [**1**-Ti(Me)(NMe(C_18_H_37_)_2_)]^+^[B(C_6_F_5_)_4_]^−^ (33 vs. 18 mol%). Another hafnium species [**4**-Hf(Me)(NMe(C_18_H_37_)_2_)]^+^[B(C_6_F_5_)_4_]^−^ showed a slightly lower yield than [**3**-Hf(Me)(NMe(C_18_H_37_)_2_)]^+^[B(C_6_F_5_)_4_]^−^ (yield 5.0 vs. 5.2 g; entry 3 vs. 2), but the yield was increased to 5.4 g, using 1.5 equiv (instead of 1.0 equiv) [Me(C_18_H_37_)_2_N-H]^+^[B(C_6_F_5_)_4_]^−^ in the activation reaction, upon which the insoluble side-product was completely dissolved. The 1-octene incorporation capability of [**4**-Hf(Me)(NMe(C_18_H_37_)_2_)]^+^[B(C_6_F_5_)_4_]^−^ was slightly better than that of [**3**-Hf(Me)(NMe(C_18_H_37_)_2_)]^+^[B(C_6_F_5_)_4_]^−^ (*F*_C8_, 34–35 vs. 33 mol%), and the *F*_C8_ values (33–35 mol%) measured for the copolymers generated with the hafnium species were comparable to or even higher than those measured for those generated with half-metallocene titanium species (24–35 mol%), which are known to be excellent for the incorporation of α-olefin. All the half-metallocene Ti species tested afforded polymers in a form dissolved in hexane.

Activated CGC-Ti species [**5**-Ti(-CH_2_N(C_18_H_37_)_2_)]^+^[B(C_6_F_5_)_4_]^−^, though it was generated uncleanly with some insoluble and soluble side-products in the overnight reaction of **5**-TiMe_2_ with 1.0 equiv [Me(C_18_H_37_)_2_N-H]^+^[B(C_6_F_5_)_4_]^−^, exhibited high activity (yield 4.9 g; entry 5), which could be further improved by mitigating the generation of insoluble side-products, using 1.5 equiv (instead of 1.0 equiv) [Me(C_18_H_37_)_2_N-H]^+^[B(C_6_F_5_)_4_]^−^ in the activation reaction (yield 6.2 g, entry 6). The species [**5**-TiCl(N(H)(C_18_H_37_)_2_)]^+^[B(C_6_F_5_)_4_]^−^ generated in the reaction of **5**-TiCl(Me) and secondary amine-derived ammonium salt [(C_18_H_37_)_2_NH_2_]^+^[B(C_6_F_5_)_4_]^−^ showed a low yield (1.2 g), which, however, could be improved by replacing trioctylaluminum (fed as a scavenger) with (iBu)_3_Al, although it was still lower than that of [**5**-Ti(CH_2_N(C_18_H_37_)_2_)]^+^[B(C_6_F_5_)_4_]^−^ (4.5 g; entry 7). For initiation of polymerization, [**5**-TiCl(N(H)(C_18_H_37_)_2_)]^+^[B(C_6_F_5_)_4_]^−^ should be alkylated (i.e., should be transformed to [**5**-TiR(N(H)(C_18_H_37_)_2_)]^+^[B(C_6_F_5_)_4_]^−^) by the action of R_3_Al, which might not be effective with bulky trioctylaluminum but occurred facilely with (iBu)_3_Al. The activated species [**6**-Ti(CH_2_N(C_18_H_37_)_2_)]^+^[B(C_6_F_5_)_4_]^−^ generated during the overnight reaction of tetrahydroquinoline-derived half-metallocene complex **6**-TiMe_2_ with [Me(C_18_H_37_)_2_N-H]^+^[B(C_6_F_5_)_4_]^−^ showed somewhat lower activity than [**5**-Ti(CH_2_N(C_18_H_37_)_2_)]^+^[B(C_6_F_5_)_4_]^−^. In this case, the yield was improved by increasing the amount of [Me(C_18_H_37_)_2_N-H]^+^[B(C_6_F_5_)_4_]^−^ used in the activation reaction (4.4, 5.3, and 5.8 g for 1.0, 1.5, and 2.0 equiv, respectively; entries 8–10). The species [**6**-Ti(N(C_18_H_37_)_2_)]^+^[B(C_6_F_5_)_4_]^−^ generated in the reaction of **6**-TiMe_2_ and secondary amine-derived ammonium salt [(C_18_H_37_)_2_NH_2_]^+^[B(C_6_F_5_)_4_]^−^ was inactive. Species [**6**-TiCl(N(H)(C_18_H_37_)_2_)]^+^[B(C_6_F_5_)_4_]^−^ generated from **6**-TiCl(Me) showed a lower yield than [**6**-Ti(CH_2_N(C_18_H_37_)_2_)]^+^[B(C_6_F_5_)_4_]^−^ (4.2 g; entry 12). The activated species [**7**-Ti(CH_2_N(C_18_H_37_)_2_)]^+^[B(C_6_F_5_)_4_]^−^ exhibited a similar performance to that of [**6**-Ti(CH_2_N(C_18_H_37_)_2_)]^+^[B(C_6_F_5_)_4_]^−^ (entries 14–15). By feeding the activated species [**7**-Ti(Me)(NMe(C_18_H_37_)_2_)]^+^[B(C_6_F_5_)_4_]^−^ as soon as it was prepared prior to transformation to [**7**-Ti(CH_2_N(C_18_H_37_)_2_)]^+^[B(C_6_F_5_)_4_]^−^, yield was improved (5.6 g vs. 4.1 g; entry 13 vs. 14). It is worth mentioning that less amounts of polymers were gained under the identical polymerization conditions when **7**-TiMe_2_ was activated with [Me(C_18_H_37_)_2_N-H]^+^[B(C_6_F_5_)_4_]^−^ that was prepared by the conventional salt metathesis method (5.6 g vs. 5.2 g, 5.1 g, 5.1 g, and 4.5 g for the ones of which ^1^H NMR spectra are shown in Figure 1a–d, respectively).

A low-molecular-weight polymer was generated with [**1**-Zr(Me)(NMe(C_18_H_37_)_2_)]^+^[B(C_6_F_5_)_4_]^−^ (*M*_w_, 32 kDa), and bimodal-molecular-weight distribution was observed with high dispersity value (*M*_w_/*M*_n_, 13) (entry 1), which inferred that structure of the active species was not persistent during the polymerization especially due to the severe temperature rising. Much-higher-molecular-weight polymers were generated from **3**- and **4**-Hf species (*M*_w_, 700–800 kDa) with much broader multimodal-molecular-weight distributions (*M*_w_/*M*_n_, 40–80), which also might be due to temperature rising (entries 2–4). From a typical CGC [**5**-Ti(CH_2_N(C_18_H_37_)_2_)]^+^[B(C_6_F_5_)_4_]^−^, polymers with b-modal-molecular-weight distributions were also generated (*M*_w_/*M*_n_, 13 and 7.3) with *M*_w_ values of 150 and 100 kDa (entries 5 and 6). However, a polymer with relatively narrow and unimodal-molecular-weight distribution was generated with [**5**-TiCl(N(H)(C_18_H_37_)_2_)]^+^[B(C_6_F_5_)_4_]^−^ (*M*_w_, 210, *M*_w_/*M*_n_, 3.6) (entry 7), which might be attributed to relatively lower polymerization temperature (90–115 °C); it has been known that the typical CGC **5**-Ti species did not exhibit thermal stability above 110 °C [27]. In contrast, half-metallocene **6**-Ti and **7**-Ti species generated polymers with unimodal-molecular-weight distributions in all cases even when polymerization temperature exceeded 120 °C (*M*_w_, 210–340 kDa; *M*_w_/*M*_n_, 2.3–3.5) (entries 8–15).

## 3. Materials and Methods

All the experiments were performed in an inert atmosphere, using a standard glove box (KOREA KIYON, Seoul, Korea) and Schlenk techniques. Toluene, hexane, diethyl ether, and THF were distilled from benzophenone ketyl. Hexane used for the polymerization reactions was purchased from Thermo Fischer Scientific (Seoul, Korea) and purified over an Na/K alloy. Sublimed-grade HfCl_4_ was purchased from Strem (Newburyport, MA, USA) and used as received. ^1^H NMR (600 MHz), ^13^C NMR (150 MHz), and ^19^F NMR (564 MHz) analyses were performed by using a JEOL ECZ 600 instrument. Elemental analyses were performed at the Analytical Center of Ajou University. The GPC data were obtained in 1,2,4-trichlorobenzene at 160 °C, using a PL-GPC 220 system equipped with an RI detector (HLC-8321 GPC/HT RI detector (Tosoh)) and two columns (PLgel mixed-B 7.5 × 300 mm from Varian (Polymer Lab)). We obtained **1**-ZrMe_2_, **2**-ZrMe_2_, and **5**-TiMe_2_ from Precious Catalysts Inc, and **3**-HfMe_2_ [52], **4**-HfMe_2_ [53], **6**-TiMe_2_ [41], and **7**-TiMe_2_ [54] were prepared according to a previously reported method.

### 3.1. Preparation of ([Me(C_18_H_37_)_2_N-H]^+^[B(C_6_F_5_)_4_]^−^, [(C_12_H_25_)_3_N-H]^+^[B(C_6_F_5_)_4_]^−^, and [(C_18_H_37_)_2_NH_2_]^+^[B(C_6_F_5_)_4_]^−^

[PhN(Me)_2_-H]^+^[B(C_6_F_5_)_4_]^−^ (2.00 g, 2.50 mmol) was added to Me(C_18_H_37_)_2_N (1.34 g, 2.50 mmol) in anhydrous toluene (20 g). After stirring overnight at room temperature, the solution was filtered over Celite to obtain a completely clear solution. The solvent was removed under vacuum. Mineral spirit (30 g, b.p., 194 °C) was added and volatiles were completely removed by distillation at 50 °C under full vacuum to obtain a clear, dark olive-green oil (3.00 g, 100%). ^1^H NMR (C_6_D_6_): δ 3.17 (br, 1H, NH), 1.97 (m, 2H, NCH_2_), 1.79 (m, 2H, NCH_2_), 1.50 (d, *J* = 4.8 Hz, 3H, NCH_3_), 1.47–1.29 (m, 48H), 1.26 (quintet, *J* = 7.8 Hz, 4H), 1.12 (quintet, *J* = 7.8 Hz, 4H), 0.94 (t, *J* = 7.2 Hz, 6H), 0.87 (quintet, *J* = 7.2 Hz, 4H), 0.80 (m, 4H) ppm. ^19^F NMR (C_6_D_6_): δ –132.22 (*ortho*-C_6_F_5_), –161.72 (t, *J* = 25.9 Hz, *para*-C_6_F_5_), −165.95 (brt, *meta*-C_6_F_5_) ppm.

[(C_12_H_25_)_3_N-H]^+^[B(C_6_F_5_)_4_]^−^ was prepared by using the same procedure and experimental conditions, using tridodecylamine (C_12_H_25_)_3_N (yield, 96%). ^1^H NMR (C_6_D_6_): δ 3.70 (br, 1H, NH), 2.19 (br, 6H, NCH_2_), 1.42–1.30 (m, 36H), 1.26 (quintet, *J* = 6.6 Hz, 6H), 1.16 (quintet, *J* = 6.0 Hz, 6H), 1.04–0.92 (m, 21H) ppm. ^19^F NMR (C_6_D_6_): δ −131.95 (*ortho*-C_6_F_5_), −161.72 (t, *J* = 21.4 Hz, *para*-C_6_F_5_), −165.91 (brt, *J* = 17.5 Hz, *meta*-C_6_F_5_) ppm. ^13^C NMR (C_6_D_6_): δ 149.05 (d, *J* = 241.2 Hz, *ortho*-C_6_F_5_), 138.98 (d, *J* = 244.2 Hz, *para*-C_6_F_5_), 137.08 (d, *J* = 247.1 Hz, *meta*-C_6_F_5_), 124.80 (br, *ipso*-C_6_F_5_), 54.39 (NCH_2_), 32.37, 30.12, 29.88 (d, *J* = 8.6 Hz), 29.67, 29.17, 26.19, 24.09, 23.15, 14.33 ppm.

[(C_18_H_37_)_2_NH_2_]^+^[B(C_6_F_5_)_4_]^−^ was also prepared by the same procedure and experimental conditions, using dioctadecylamine (C_18_H_37_)_2_NH (yield, 100%). ^1^H NMR (C_6_D_6_): δ 3.11 (br, 2H, NH_2_), 1.80 (br, 4H, NCH_2_), 1.47–1.26 (m, 48H), 1.22 (quintet, *J* = 7.2 Hz, 4H), 1.10 (quintet, *J* = 7.2 Hz, 4H), 0.93 (t, *J* = 7.2 Hz, 6H), 0.84 (quintet, *J* = 7.2 Hz, 4H), 0.74 (m, 4H) ppm. ^19^F NMR (C_6_D_6_): δ −132.16 (*ortho*-C_6_F_5_), −161.70 (t, *J* = 22.0 Hz, *para*-C_6_F_5_), −165.97 (brt, *J* = 17.5 Hz, *meta*-C_6_F_5_) ppm. ^13^C NMR (C_6_D_6_): δ 149.05 (d, *J* = 238.5 Hz, *ortho*-C_6_F_5_), 138.96 (d, *J* = 245.7 Hz, *para*-C_6_F_5_), 137.10 (d, *J* = 248.5 Hz, *meta*-C_6_F_5_), 124.73 (br, *ipso*-C_6_F_5_), 49.52 (NCH_2_), 32.39, 30.25 (m), 29.89 (d, *J* = 5.7 Hz), 29.63, 29.05, 25.95 (d, *J* = 11.4 Hz), 23.15, 14.35 ppm.

### 3.2. Preparation of 4-HfMe_2_ and 3-HfMe_2_

First, nBuLi (1.07 mL, 2.5 M in hexane, 2.68 mmol) was diluted with hexane (1.4 mL) and added dropwise to a stirred suspension of 2,7-di-*tert*-butyl-9-(cyclopentadienyldiphenylmethyl)-9*H*-fluorene (**4**-H_2_, 0.661 g, 1.30 mmol) in diethyl ether (28 mL) at −78 °C. After the suspension was stirred overnight at room temperature, the solvent was removed, using a vacuum line. The residue was washed with hexane (20 mL) to obtain a light red solid (0.643 g, 95%), which was identified as **4**-Li_2_ by ^1^H NMR analysis. The isolated solid (0.784 g, 1.51 mmol) was dissolved in THF (14 mL), cooled at −30 °C, and MeMgBr (1.00 g, 3.28 M in diethyl ether; density = 1.035 g/mL, 3.17 mmol) and HfCl_4_ (0.473 g, 1.48 mmol) were successively added. After stirring overnight at room temperature, the solvent was removed, using a vacuum line. The residue was extracted by using hot hexane (35 mL). The filtrate was allowed to stand at −30 °C for 1 d to deposit yellow crystals that were analytically pure and suitable for X-ray crystallography (0.45 g, 41%). ^1^H NMR (C_6_D_6_): δ 8.03 (d, *J* = 8.4 Hz, 2 H), 7.89 (d, *J* = 7.8 Hz, 2H), 7.70 (d, *J* = 7.8 Hz, 2H), 7.45 (dd, *J* = 8.4, 1.8 Hz, 2H), 7.13 (td, *J* = 7.8, 1.2 Hz, 2H), 7.05 (td, *J* = 7.8, 1.2 Hz, 2H), 6.95 (t, *J* = 7.8 Hz, 2H), 6.45 (s, 2H), 6.11 (t, *J* = 3.0 Hz, 2H), 5.56 (t, *J* = 3.0 Hz, 2H), 1.11 (s, 18H), −1.35 (s, Hf(CH_3_)_2_, 6H) ppm. ^13^C NMR (C_6_D_6_): δ 148.69, 146.44, 130.15, 128.84, 128.70, 127.03, 126.70, 123.86, 123.28, 121.42, 119.31, 116.76, 112.22, 108.21, 101.99, 77.13, 58.34, 38.91, 35.09, 31.12 ppm. Anal. Calcd. (C_41_H_44_Hf): C, 68.85; H, 6.20%. Found: C, 68.47; H, 6.50%.

Complex **3** was prepared by using the same procedure and experimental conditions with **3**-H_2_. ^1^H NMR (C_6_D_6_): δ 8.40 (d, *J* = 1.8 Hz, 2 H), 7.87 (d, *J* = 7.8 Hz, 2H), 7.77 (d, *J* = 8.4 Hz, 2H), 7.15 (td, *J* = 7.8, 1.4 Hz, 2H), 7.03 (td, *J* = 6.0, 1.4 Hz, 2H), 6.97 (t, *J* = 7.2 Hz, 2H), 6.85 (dd, *J* = 9.3, 1.8 Hz, 2H), 6.41 (d, *J* = 9.6 Hz, 2H), 6.10 (t, *J* = 2.4 Hz, 2H), 5.47 (t, *J* = 3.0 Hz, 2H), 1.35 (s, 18H), −1.37 (s, Hf(CH_3_)_2_, 6H) ppm. ^13^C NMR (C_6_D_6_): δ 146.81, 146.55, 130.13, 128.97, 128.74, 127.28, 126.79, 126.09, 123.27, 121.98, 118.94, 118.78, 112.32, 109.04, 102.78, 77.52, 58.73, 39.15, 34.98, 32.04 ppm. Anal. Calcd. (C_41_H_44_Hf): C, 68.85; H, 6.20%. Found: C, 68.56; H, 6.42%.

### 3.3. Preparation of 5-TiCl(Me) and 6-TiCl(Me)

ZnCl_2_ (20.8 mg, 0.153 mmol) was added to **5**-TiMe_2_ (100 mg, 0.305 mmol) in anhydrous toluene (2 mL). Insoluble ZnCl_2_ gradually disappeared, and an almost-clear solution was obtained after stirring for several hours at room temperature. After removing cloudy, insoluble fractions via filtration, the solvent was removed, using a vacuum line, to obtain a yellow solid. An analytically pure compound was obtained by recrystallization in hexane at −30 °C. Yellow cubic-shaped crystals suitable for X-ray crystallography were isolated (80 mg, 76%). ^1^H NMR (C_6_D_6_): δ 2.01 (s, 3H), 1.96 (s, 3H), 1.92 (s, 3H), 1.90 (s, 3H), 1.51 (s, 9H), 0.84 (s, 3H), 0.46 (s, 3H), 0.38 (s, 3H) ppm. ^13^C NMR (C_6_D_6_): δ 136.49, 134.56, 133.43, 132.72, 101.56, 59.59, 53.31, 33.90, 15.48, 15.11, 12.68, 12.11, 5.86 (d, *J* = 5.7 Hz) ppm. Anal. Calcd. (C_16_H_30_ClNSiTi): C, 55.25; H, 8.69; N, 4.03%. Found: C, 55.54; H, 8.91; N, 3.73%.

Using **6**-TiMe_2_, **6**-TiCl(Me) was prepared with the same procedure and experimental conditions. Red needle-shaped crystals were isolated (90 mg, 85%). ^1^H NMR (C_6_D_6_): δ 6.98 (m, 1H), 6.88 (m, 2H), 4.72 (ddd, *J* = 7.2, 3.6 Hz, 1H), 4.28 (ddd, *J* = 8.4, 3.0 Hz, 1H), 2.31 (t, *J* = 6.6 Hz, 2H), 2.04 (s, 3H), 1.95 (s, 3H), 1.77 (s, 3H), 1.62 (s, 3H), 1.54 (m, 2H), 0.90 (s, 3H) ppm. ^13^C NMR (C_6_D_6_): δ 161.68, 139.63, 137.07, 136.91, 129.11, 128.92, 126.72, 124.78, 123.90, 121.44, 120.29, 54.40, 51.04, 26.71, 22.51, 12.48, 12.22, 11.91, 11.85 ppm. Anal. Calcd. (C_19_H_24_ClNTi): C, 65.25; H, 6.92; N, 4.01%. Found: C, 65.53; H, 7.30; N, 3.75%.

### 3.4. A Representative Activation Reaction

First **4**-HfMe_2_ (7.15 mg, 10.0 µmol) dissolved in C_6_D_12_ was added to a solution of [Me(C_18_H_37_)_2_N-H]^+^[B(C_6_F_5_)_4_]^−^ (12.2 mg, 10.0 µmol) in C_6_D_12_ in an NMR tube. After sealing the tube, the activation reaction was monitored, using ^1^H NMR spectroscopy. After finishing the ^1^H NMR studies, the solution was completely transferred to a vial and diluted with cyclohexane to obtain 5.29 µmol-Hf/g stock solution, which was used in the polymerization studies.

### 3.5. A Representative Polymerization Procedure (Entry 4)

In a glove box, a dried bomb reactor (75 mL) was charged with hexane (15.5 g), 1-octene (5.00 g), and trioctylaluminum (12.1 g, 33.0 µmol). The reactor was then assembled and removed from the glove box. The bomb reactor was immersed in a bath at 110 °C. When the temperature inside the bomb reactor reached 65 °C, a catalyst stock solution containing [**4**-Hf(Me)(NMe(C_18_H_37_)_2_)]^+^[B(C_6_F_5_)_4_]^−^ (1.00 µmol) was injected. After catalyst feeding, ethylene gas was immediately charged under a pressure of 20 bar. The temperature immediately started to rise, reaching 159 °C in 3 min. The polymerization was performed for 15 min under a constant ethylene pressure of 20 bar while monitoring the temperature in an isothermal bath at 110 °C. In some cases (entries 2–3, 7–10, and 14), polymerization was not initiated until temperature reaching some threshold, in which cases polymerization reaction was performed for 15 min counted from the initiation point. The reactor was cooled with an ice bath and the ethylene gas was vented off. The generated polymer was isolated by removing the solvent under vacuum at 130 °C for 2 h (5.41 g).

### 3.6. X-ray Crystallography

Specimens of suitable quality and size were selected, mounted, and centered in the X-ray beam, using a video camera. Reflection data were collected at 100 K on an APEX II CCD area diffractometer (Bruker), using graphite-monochromated Mo Kα radiation (λ = 0.7107 Ǻ). The hemisphere of the reflection data was collected as φ and ω scan frames at 0.5° per frame and an exposure time of 10 s per frame. The cell parameters were determined and refined by using the SMART program. Data reduction was performed by using SAINT software. The data were corrected for Lorentz and polarization effects. Empirical absorption correction was applied by using the SADABS program. The structure was solved by using direct methods and refined with the full matrix least-squares method, using the SHELXTL package and the olex2 program with anisotropic thermal parameters for all non-hydrogen atoms.

The crystallographic data for **4**-HfMe_2_⋅0.4(toluene) (CCDC# 2070013) that were used in all calculations were as follows: C_87.6_H_94.4_Hf_2_, M = 1504.21, triclinic, *a* = 15.1760(2), *b* = 15.9555(2), *c* = 17.8025(3) Å, *α* = 63.5766(6)°, *β* = 69.4527(8)°, *γ* = 81.8613(7), *V* = 3614.24(9) Å^3^, space group *P*-1, Z = 2, ρ_calc_ (g/cm^3^) = 1.382, μ(mm^−1^) = 2.914, F(000) = 1528, number of reflections collected = 49,830, restraints = 178, parameters = 869, and number of unique reflections = 13,827 (R(int) = 0.0605). The final GOF, *R*_1_, and *wR*_2_ were 1.032, 0.0400, and 0.0828 (I > 2σ(I)), respectively.

The crystallographic data for **5**-TiCl(Me) (CCDC# 2070015) that were used in all calculations were as follows: C_16_H_30_ClNSiTi, *M* = 347.82, monoclinic, *a* = 13.3706(9), *b* = 12.1586(8), *c* = 11.5983(8) Å, β = 90.542(2)°, *V* = 1885.4 (2) Å^3^, space group *P*2_1_/c, *Z* = 4, ρ_calc_ (g/cm^3^) = 1.225, μ(mm^−1^) = 0.651, F(000) = 744, number of reflections collected = 19,653, restraints = 30, parameters = 242, and number of unique reflections = 3569 (R(int) = 0.0827). The final GOF, *R*_1_, and *wR*_2_ were 1.070, 0.0520, and 0.1335 (I > 2σ(I)), respectively.

The crystallographic data for **6**-TiCl(Me) (CCDC# 2070014) that were used in all calculations were as follows: C_19_H_24_ClNTi, *M* = 349.56, orthorhombic, *a* = 13.3739(3), *b* = 17.6098(3), *c* = 14.9636(3) Å, *V* = 3524.10(12) Å^3^, space group *P*bca, *Z* = 8, ρ_calc_ (g/cm^3^) = 1.318, μ(mm^−1^) = 0.634, F(000) = 1472, number of reflections collected = 44,800, restraints = 12, parameters = 225, and number of unique reflections = 3348 (R(int) = 0.1804). The final GOF, *R*_1_, and *wR*_2_ were 1.031, 0.0587, and 0.1179 (I > 2σ(I)), respectively.

## 4. Conclusions

High-purity tertiary or secondary ammonium tetrakis(pentafluorophenyl)borate ([Me(C_18_H_37_)_2_N-H]^+^[B(C_6_F_5_)_4_]^−^, [(C_12_H_25_)_3_N-H]^+^[B(C_6_F_5_)_4_]^−^, and [(C_18_H_37_)_2_NH_2_]^+^[B(C_6_F_5_)_4_]^−^) containing neither water nor Cl^−^ salt impurities were prepared by the acid–base reaction of [PhN(Me)_2_-H]^+^[B(C_6_F_5_)_4_]^−^ with the corresponding amine. The action of [Me(C_18_H_37_)_2_N-H]^+^[B(C_6_F_5_)_4_]^−^ on typical ansa-metallocene complexes *rac*-[ethylenebis(tetrahydroindenyl)]Zr(Me)_2_ (**1**-ZrMe_2_), [Ph_2_C(Cp)(3,6-*^t^*Bu_2_Flu)]Hf(Me)_2_ (**3**-HfMe_2_), and ([Ph_2_C(Cp)(2,7-*^t^*Bu_2_Flu)]Hf(Me)_2_ (**4**-HfMe_2_) afforded clean [**L**-M(Me)(NMe(C_18_H_37_)_2_)]^+^[B(C_6_F_5_)_4_]^−^-type complexes. In contrast, the corresponding species formed in the reaction of half-metallocene titanium complexes [(η^5^-Me_4_C_5_)Si(Me)_2_(κ-N^t^Bu)]Ti(Me)_2_ (**5**-TiMe_2_), [(η^5^-Me_4_C_5_)(C_9_H_9_(κ-N))]Ti(Me)_2_ (**6**-TiMe_2_), and [(η^5^-Me_3_C_7_H_1_S)(C_10_H_11_(κ-N))]Ti(Me)_2_ (**7**-TiMe_2_) with [Me(C_18_H_37_)_2_N-H]^+^[B(C_6_F_5_)_4_]^−^ were unstable for subsequent transformation to other species (presumably, [**L**-Ti(CH_2_N(C_18_H_37_)_2_)]^+^[B(C_6_F_5_)_4_]^−^-type complexes). The addition of excess (1.5 equiv.) [Me(C_18_H_37_)_2_N-H]^+^[B(C_6_F_5_)_4_]^−^ suppressed the formation of side products, especially in the cases of **4**-HfMe_2_, **5**-TiMe_2_, **6**-TiMe_2_, and **7**-TiMe_2_. With the aim of preventing further transformation, **5**-TiCl(Me) and **6**-TiCl(Me) were prepared, from which [**L**-TiCl(N(H)(C_18_H_37_)_2_)]^+^[B(C_6_F_5_)_4_]^−^-type complexes were successfully prepared. The [**L**-M(Me)(NMe(C_18_H_37_)_2_)]^+^[B(C_6_F_5_)_4_]^−^, [**L**-Ti(CH_2_N(C_18_H_37_)_2_)]^+^[B(C_6_F_5_)_4_]^−^, and [**L**-TiCl(N(H)(C_18_H_37_)_2_)]^+^[B(C_6_F_5_)_4_]^−^-type species, which are soluble and stable in aliphatic hydrocarbon solvents (e.g., methylcyclohexane and C_6_D_12_), were highly active in ethylene/1-octene copolymerization performed in aliphatic hydrocarbon solvents (e.g., hexane). Feeding the activated species to a polymerization reactor (instead of in situ generation of the activated species in a bulk reactor) might be advantageous in running a commercial process.

## 5. Patents

Patents were applied on this study (Ajou University, high-purity ammonium borate and a method for preparing the same, Kr 10-2020-0104473, 20 August 2020; Ajou University, halogenation of group 4 dimethyl olefin polymerization catalysts, Kr 10-2021-0045949, 08 April 2021).

## Figures and Tables

**Table 1:** Polymerization results ^a^.

Entry	Catalyst	Temperature ^b^ (°C)	Yield (g)	*F*_Oct_^c^ (mol%)	*M*_w_^d^ (kDa)	*M*_w_/*M*_n_
1	[**1**-Zr(Me)(NMe(C_18_H_37_)_2_)]^+^[B(C_6_F_5_)_4_]^−^	65–116–94	9.3	18	32	13
2	[**3**-Hf(Me)(NMe(C_18_H_37_)_2_)]^+^[B(C_6_F_5_)_4_]^−^	82–164–91	5.2	33	716	39
3	[**4**-Hf(Me)(NMe(C_18_H_37_)_2_)]^+^[B(C_6_F_5_)_4_]^−^	78–161–91	5.0	34	814	56
4 ^e^	[**4**-Hf(Me)(NMe(C_18_H_37_)_2_)]^+^[B(C_6_F_5_)_4_]^−^	65–159–95	5.4	35	696	78
5	[**5**-Ti(CH_2_N(C_18_H_37_)_2_)]^+^[B(C_6_F_5_)_4_]^−^	65–141–82	4.9	35	150	13
6 ^e^	[**5**-Ti(CH_2_N(C_18_H_37_)_2_)]^+^[B(C_6_F_5_)_4_]^−^	65–126–83	6.2	29	103	7.3
7 ^f^	[**5**-TiCl(N(H)(C_18_H_37_)_2_)]^+^[B(C_6_F_5_)_4_]^−^	89–115–99	4.5	25	211	3.6
8	[**6**-Ti(CH_2_N(C_18_H_37_)_2_)]^+^[B(C_6_F_5_)_4_]^−^	81–102–95	4.4	27	269	2.3
9 ^e^	[**6**-Ti(CH_2_N(C_18_H_37_)_2_)]^+^[B(C_6_F_5_)_4_]^−^	80–124–91	5.3	29	237	2.5
10 ^g^	[**6**-Ti(CH_2_N(C_18_H_37_)_2_)]^+^[B(C_6_F_5_)_4_]^−^	71–129–83	5.8	31	211	3.5
11	[**6**-Ti(N(C_18_H_37_)_2_)]^+^[B(C_6_F_5_)_4_]^−^	-	0	-		
12 ^f^	[**6**-TiCl(N(H)(C_18_H_37_)_2_)]^+^[B(C_6_F_5_)_4_]^−^	65–129–82	4.2	32	213	2.7
13 ^h^	[**7**-Ti(Me)(NMe(C_18_H_37_)_2_)]^+^[B(C_6_F_5_)_4_]^−^	65–104–79	5.6	35	209	2.9
14	[**7**-Ti(CH_2_N(C_18_H_37_)_2_)]^+^[B(C_6_F_5_)_4_]^−^	80–104–94	4.1	24	343	2.5
15 ^e^	[**7**-Ti(CH_2_N(C_18_H_37_)_2_)]^+^[B(C_6_F_5_)_4_]^−^	65–119–85	5.3	29	216	3.4

^a^ Polymerization conditions: **L**-MMe_2_ (1.0 μmol) activated with [Me(C_18_H_37_)_2_N-H]^+^[B(C_6_F_5_)_4_]^−^ (1.0 or 1.5 equiv) or **L**-MCl(Me) (1.0 μmol) activated with [(C_18_H_37_)_2_NH_2_]^+^[B(C_6_F_5_)_4_]^−^ (1.1 equiv), trioctylaluminum (33 μmol) as a scavenger, hexane (15.5 g), 1-octene (5.0 g), ethylene (20 bar), 15 min. ^b^ Temperature was not controlled (temperature starting to rise/the highest temperature reached after several minutes/temperature at the end of polymerization). ^c^ 1-Octene content measured by ^1^H-NMR spectra. ^d^ Measured by GPC at 160 °C, using trichlorobenzene and calculated relative to PS standards. ^e^ A 1.5 equiv [Me(C_18_H_37_)_2_N-H]^+^[B(C_6_F_5_)_4_]^−^ was used. ^f^ (iBu)_3_Al was used instead of trioctylaluminum. ^g^ A 2.0 equiv [Me(C_18_H_37_)_2_N-H]^+^[B(C_6_F_5_)_4_]^−^ was used. ^h^ The activation reaction time was short (<3 min).

## Data Availability

The data that support the findings of this study are available from the corresponding author upon reasonable request.

## Supplementary Materials

The following are available online. Figure S1: Ag^+^ ion test results for [(Me)(C_18_H_37_)_2_N-H]^+^[B(C_6_F_5_)_4_]^−^; Figure S2: ^1^H NMR spectrum (recorded in C_6_D_6_) for the sample in which water was deliberately added to the high purity [(Me)(C_18_H_37_)_2_N-H]^+^[B (C_6_F_5_)_4_]^−^ prepared according to Scheme 2b; Figures S3 and S4: ^1^H, ^13^C, and ^19^F NMR spectra of [(C_12_H_25_)_3_N-H]^+^[B(C_6_F_5_)_4_]^−^ and [(C_18_H_37_)_2_NH_2_]^+^[B(C_6_F_5_)_4_]^−^ recorded in C_6_D_6_; Figure S5: ^1^H NMR spectra of **6**-TiCl(Me) and its activated complex [**6**-TiCl(N(H)(CF_18_H_37_)_2_)]^+^[B(C_6_F_5_)_4_]^−^; Figure S6: ^1^H and ^13^C NMR spectra of **6**-TiCl_2_ generated by reacting **6**-TiMe_2_ with 1 eq ZnCl_2_; Figures S7–S10: ^1^H-^1^H COSY, ^13^C, ^1^H-^13^C HSQC, and ^19^F NMR spectrum of [**1**-Zr(Me)(N(Me)(C_18_H_37_)_2_)]^+^[B(C_6_F_5_)_4_]^−^; Figure S11: ^1^H NMR spectra of Me(C_18_H_37_)_2_N and [(Me)(C_18_H_37_)_2_N-H]^+^[B(C_6_F_5_)_4_]^−^ recorded in C_6_D_12_; Figure S12: ^1^H NMR spectra of **4**-HfMe_2_ and its activated complex [**4**-Hf(Me)(N(Me)(C_18_H_37_)_2_)]^+^[B(C_6_F_5_)_4_]^−^; Figure S13: ^1^H NMR spectra of **5**-TiMe_2_ and its reaction product with [(Me)(C_18_H_37_)_2_N-H]^+^[B(C_6_F_5_)_4_]^−^; Figure S14: ^1^H NMR spectra for the reaction of **6**-TiMe_2_ with [(C_18_H_37_)_2_NH_2_]^+^[B(C_6_F_5_)_4_]^−^ in 10 min and in 2 h; Figure S15: ^1^H NMR spectra of **7**-TiMe_2_ and its activated complex [**7**-Ti(η^1^-CH_2_)N(C_18_H_37_)_2_]^+^[B(C_6_F_5_)_4_]^−^ formed at an initial stage which was transformed to another species after overnight.

## Sample Availability

Samples of [(Me)(C_18_H_37_)_2_N-H]^+^[B(C_6_F_5_)_4_]^−^, [(C_12_H_25_)_3_N-H]^+^[B(C_6_F_5_)_4_]^−^, and [(C_18_H_37_)_2_NH_2_]^+^[B(C_6_F_5_)_4_]^−^ are available from Precious Catalysts Inc.; cglee@s-pci.com (C.G.L.).
