# Supplementary material for: Preparation of High-Purity Ammonium Tetrakis(pentafluorophenyl)borate for the Activation of Olefin Polymerization Catalysts
Source: Molecules. 2021 May 10;26(9):2827. doi: 10.3390/molecules26092827 (PMC8126221; doi:10.3390/molecules26092827)

Supporting Information: Preparation of High-Purity Ammonium Tetrakis(pentafluorophenyl)borate for the Activation of Olefin Polymerization Catalysts

Hyun Ju Lee [1,†], Jun Won Baek [1,†], Yeong Hyun Seo [1], Hong Cheol Lee [1], Sun Mi Jeong [1], Junseong Lee [2], Chong Gu Lee [3], and Bun Yeoul Lee [1,*]

[1]Department of Molecular Science and Technology, Ajou University, Suwon 16499, South Korea; hjulee4639@ajou.ac.kr (H.J.L.); btw91@ajou.ac.kr (J.W.B.); tdg0730@ajou.ac.kr (Y.H.S.); asdfg7235950@gmail.com (H.C.L.); sunmi7523@ajou.ac.kr (S.M.J.)
[2]Department of Chemistry, Chonnam National University, 77 Yongbong-ro, Buk-gu, Gwangju 61186, South Korea; leespy@chonnam.ac.kr (J.L.)
[3]Precious Catalysts Inc., 201 Duryu-gil, Angangeup, Gyeongju 38029, South Korea; cglee@s-pci.com (C.G.L.)
*Correspondence: bunyeoul@ajou.ac.kr; Tel: 82-31-219-1844
[†]These authors contributed equally to this work.

Figure S1. Ag^+ ion test results for $[(Me)(C_{18}H_{37})_2N\text{-}H]^+[B(C_6F_5)_4]^-$ prepared by the reactions of (**a**) "$[PhN(Me)_2\text{-}H]^+[B(C_6F_5)_4]^-$ + $(Me)(C_{18}H_{37})_2N$", (**b**) "$Li^+[B(C_6F_5)_4]^-$ + $[(Me)(C_{18}H_{37})_2N\text{-}H]^+Cl^-$ (removal of LiCl by water)", (**c**) "$Li^+[B(C_6F_5)_4]^-$ + $[(Me)(C_{18}H_{37})_2N\text{-}H]^+Cl^-$ (removal of LiCl by filtration)", (**d**) "$K^+[B(C_6F_5)_4]^-$ + $[(Me)(C_{18}H_{37})_2N\text{-}H]^+Cl^-$ (removal of KCl by water)", and (**e**) "$Li^+[B(C_6F_5)_4]^-$ + $[(Me)(C_{18}H_{37})_2N\text{-}H]^+Cl^-$ (removal of KCl by filtration)".

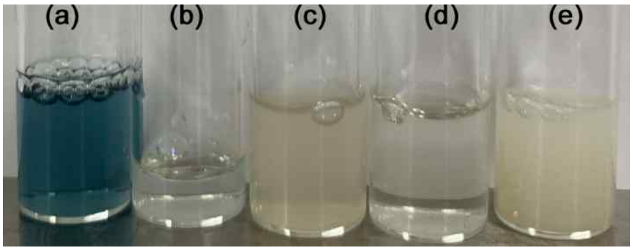

Figure S2. ^1H NMR spectrum (recorded in C_6D_6) for the sample in which water was deliberately added to the high purity $[(Me)(C_{18}H_{37})_2N\text{-}H]^+[B\ (C_6F_5)_4]^-$ prepared according to Scheme 2b.

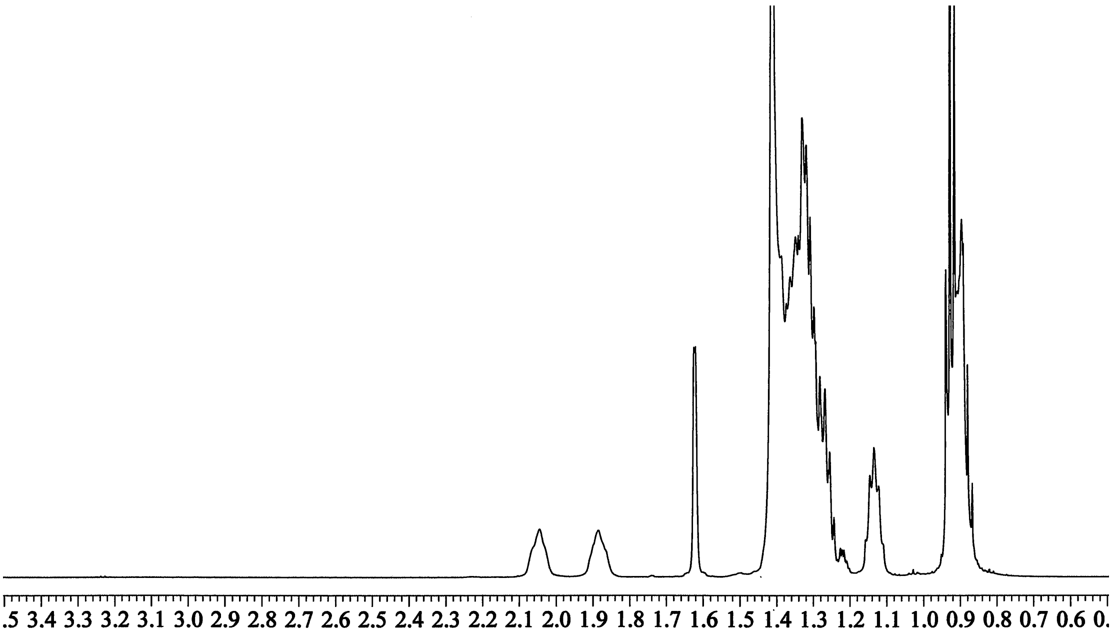

Figure S3. ^1H, ^{13}C, and ^{19}F NMR spectra of $[(C_{12}H_{25})_3N\text{-}H]^+[B(C_6F_5)_4]^-$ recorded in C_6D_6.

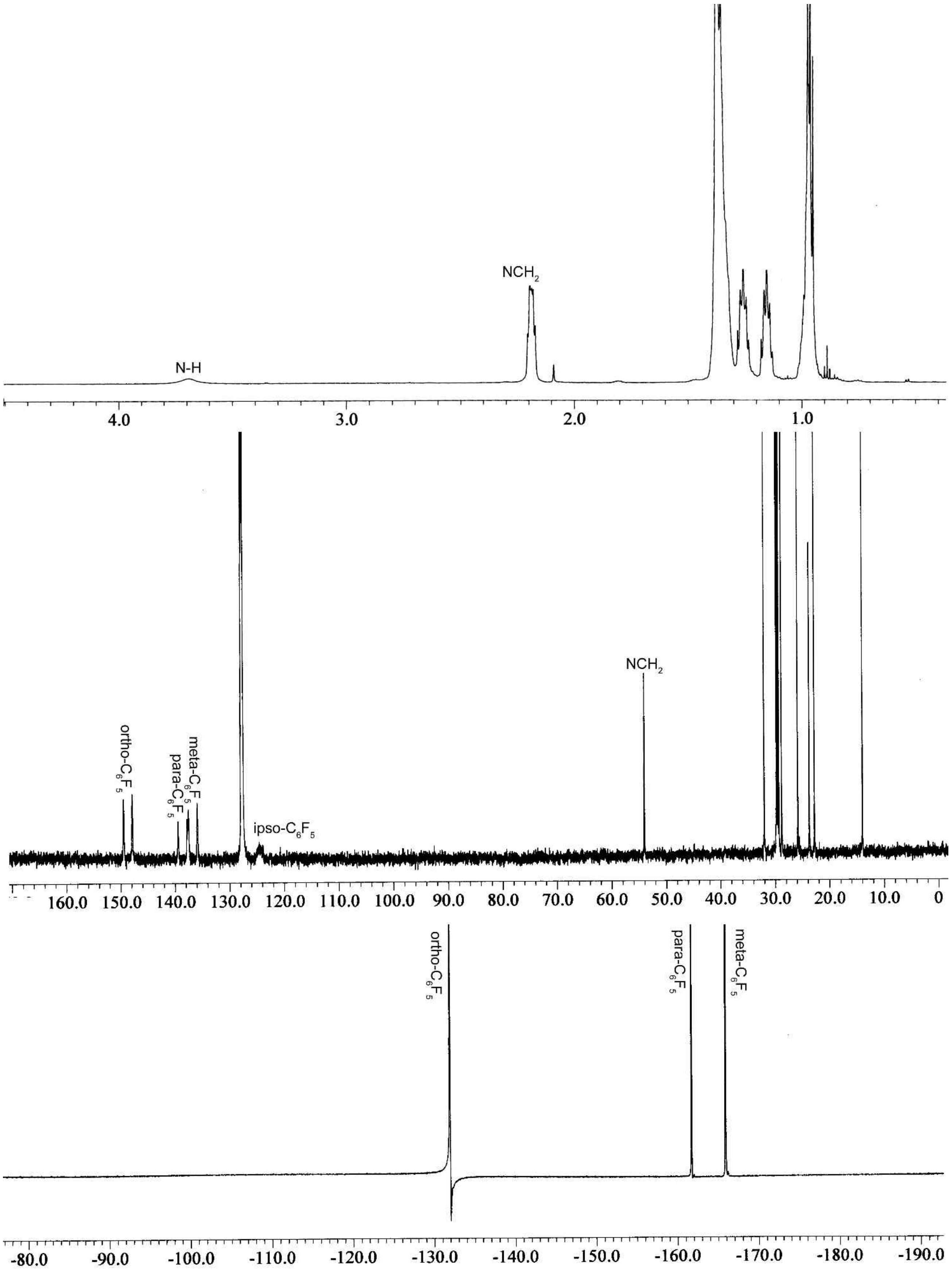

Figure S4. ^{1}H, ^{13}C, and ^{19}F NMR spectra of $[(C_{18}H_{37})_2NH_2]^+[B(C_6F_5)_4]^-$ recorded in C_6D_6.

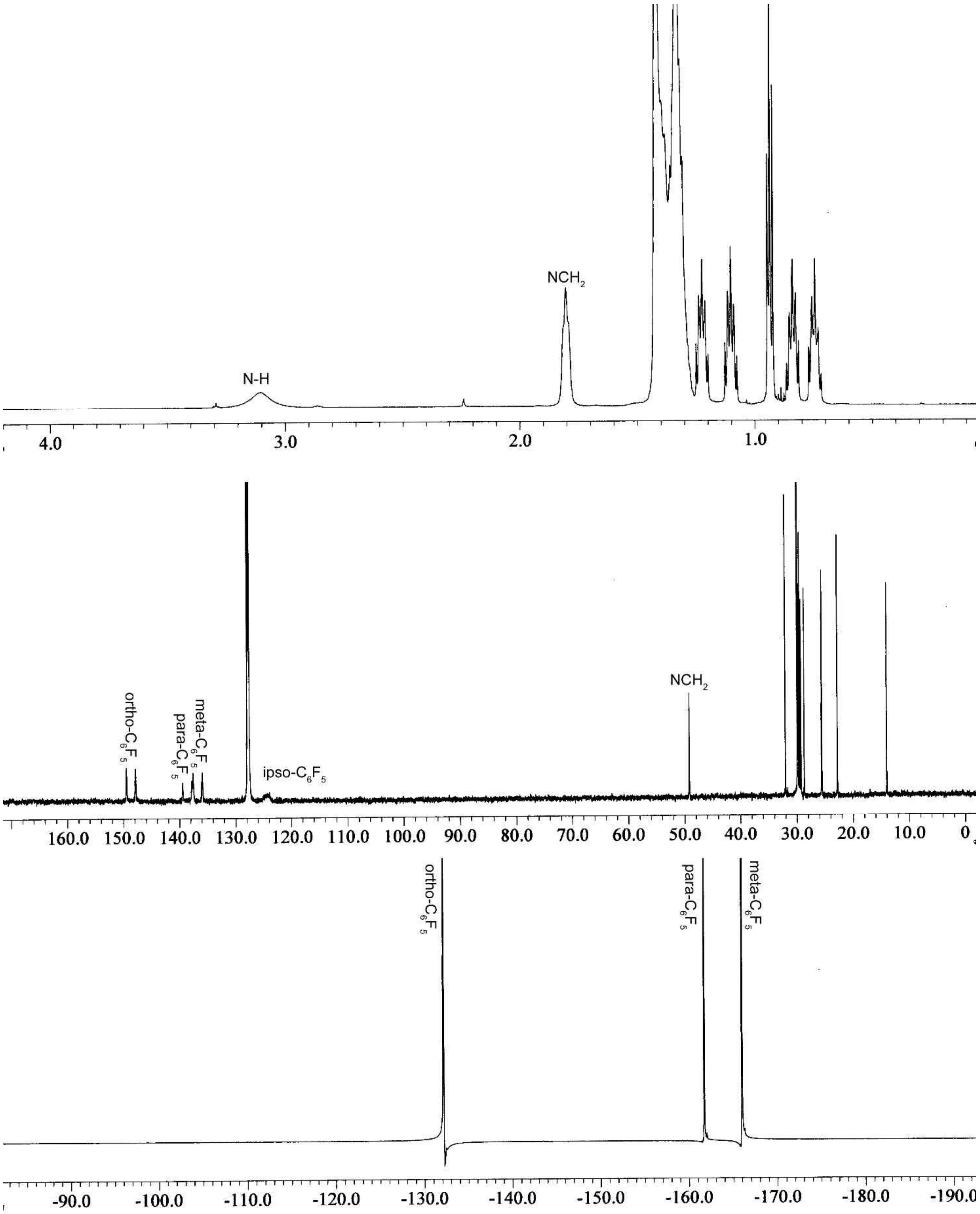

Figure S5. ^{1}H NMR spectra of **6**-TiCl(Me) (**a**) and its activated complex [**6**-TiCl(N(H)($C_{18}H_{37}$)$_2$)]$^+$[B(C_6F_5)$_4$]$^-$ (**b**).

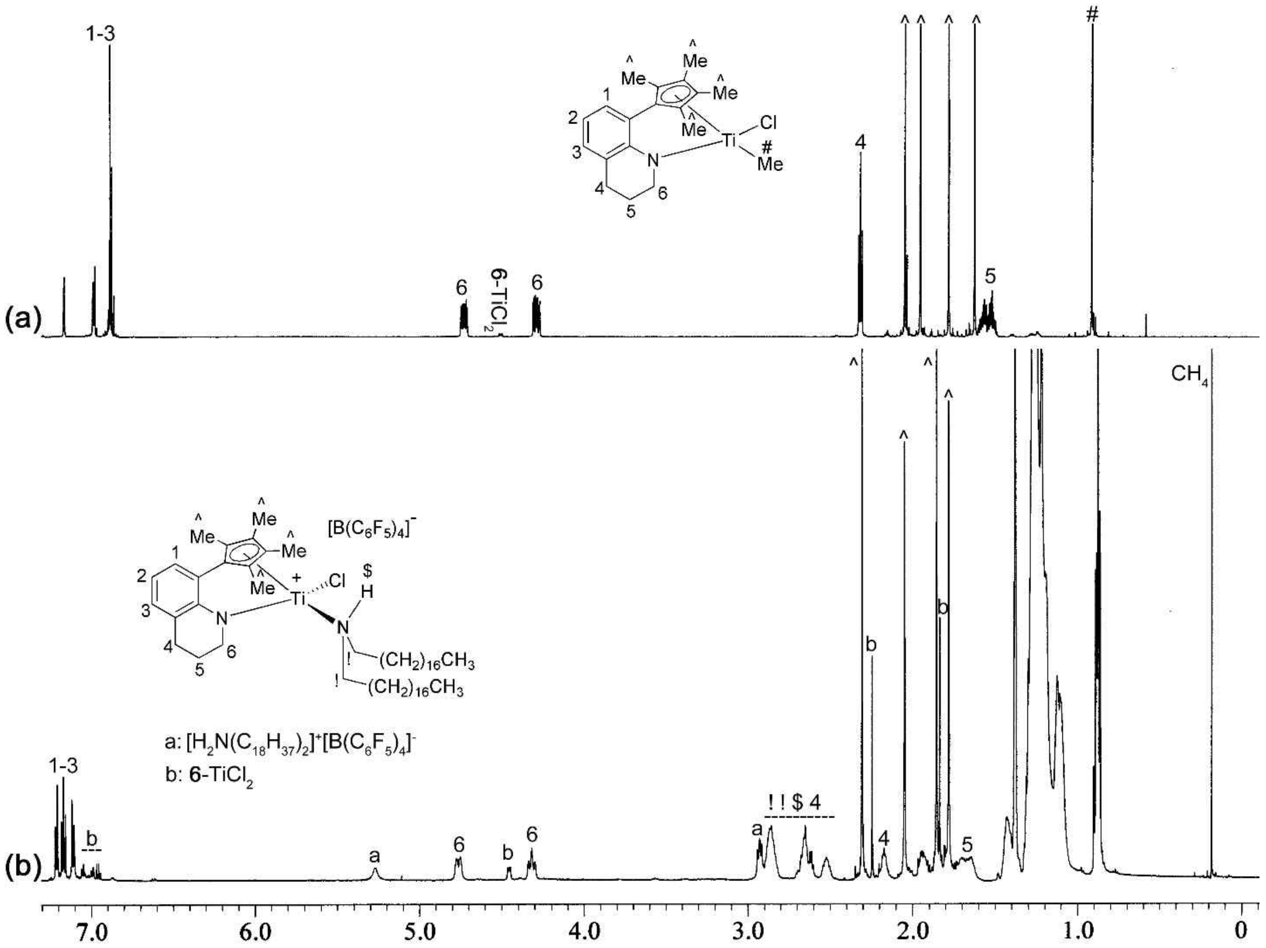

Figure S6. ^{1}H and ^{13}C NMR spectra of **6**-$TiCl_2$ generated by reacting **6**-$TiMe_2$ with 1 eq $ZnCl_2$.

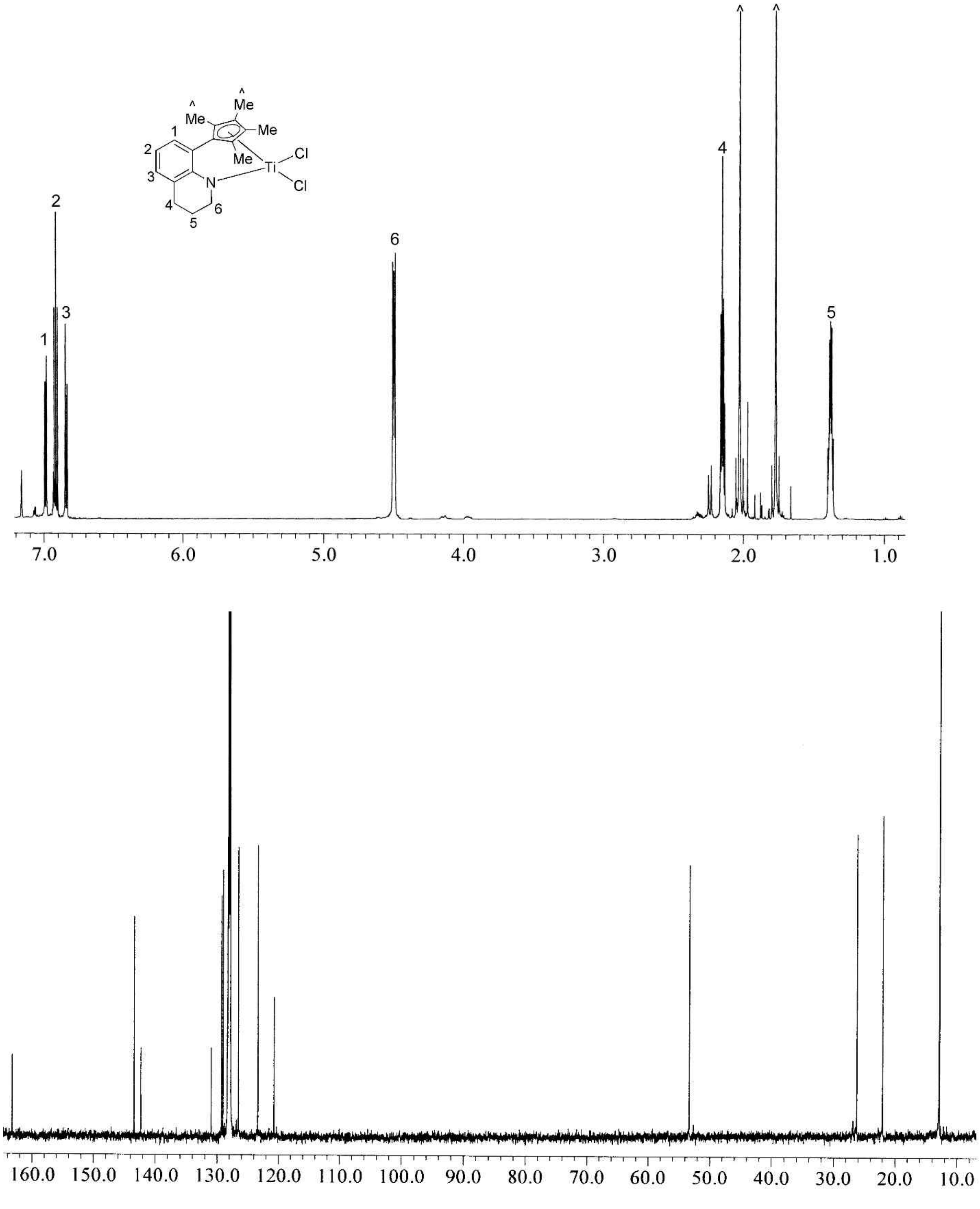

Figure S7. ^{1}H-^{1}H COSY NMR spectrum of [**1**-Zr(Me)(N(Me)($C_{18}H_{37}$)$_2$)]$^+$[B(C_6F_5)$_4$]$^-$.

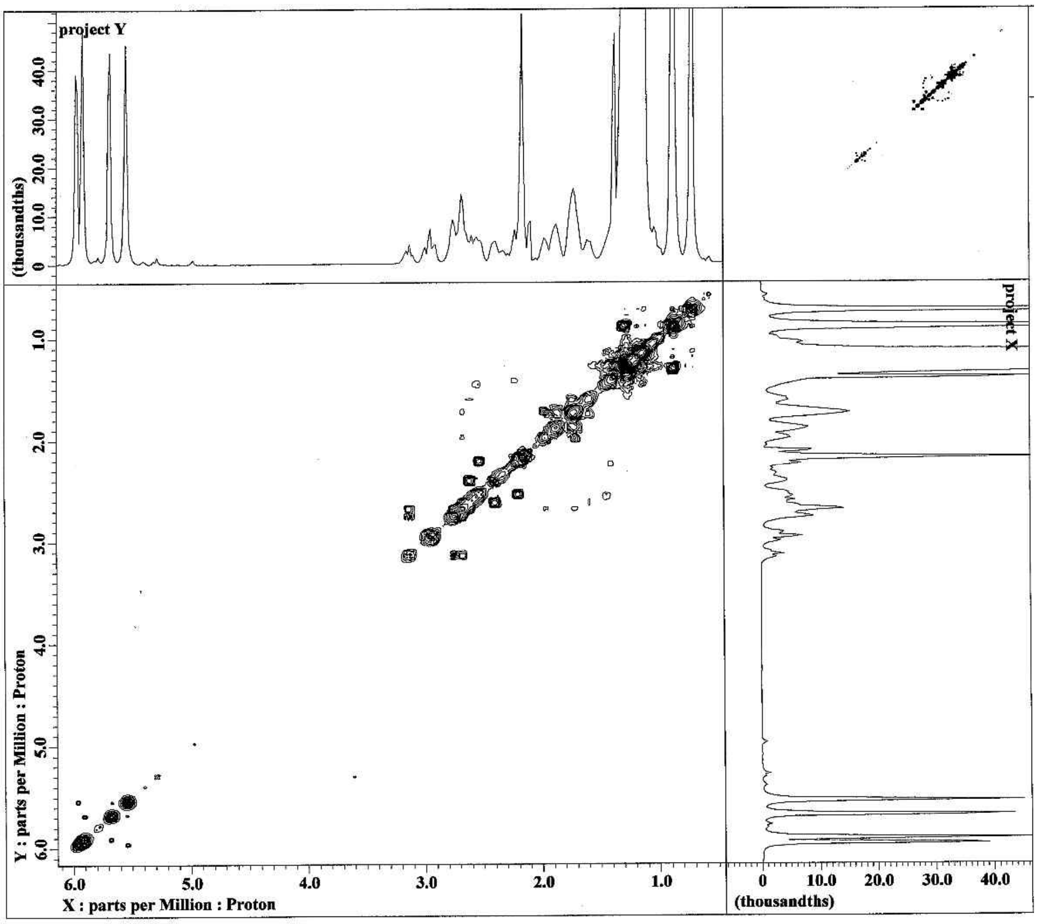

Figure S8. ^{13}C NMR spectrum of [**1**-Zr(Me)(N(Me)($C_{18}H_{37}$)$_2$)]$^+$[B(C_6F_5)$_4$]$^-$.

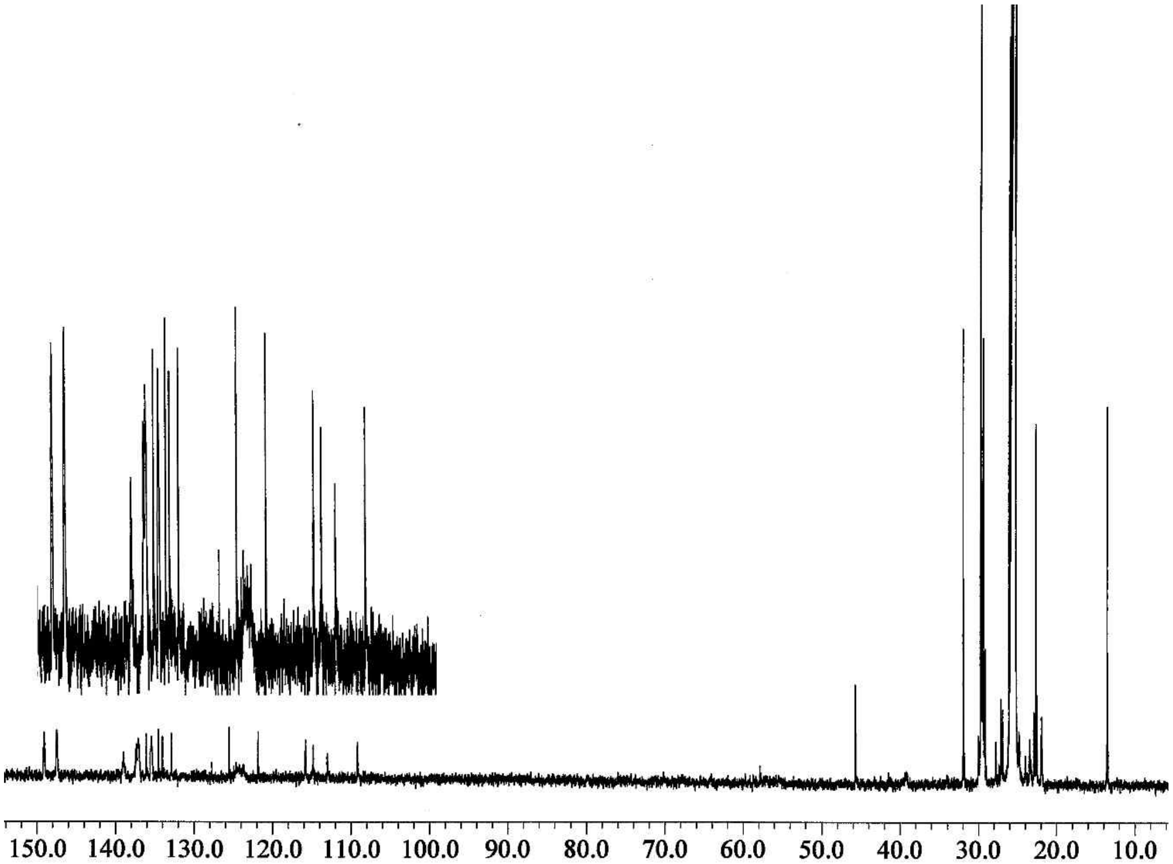

Figure S9. ^{1}H-^{13}C HSQC NMR spectrum of [**1**-Zr(Me)(N(Me)($C_{18}H_{37}$)$_2$)]$^+$[B(C_6F_5)$_4$]$^-$.

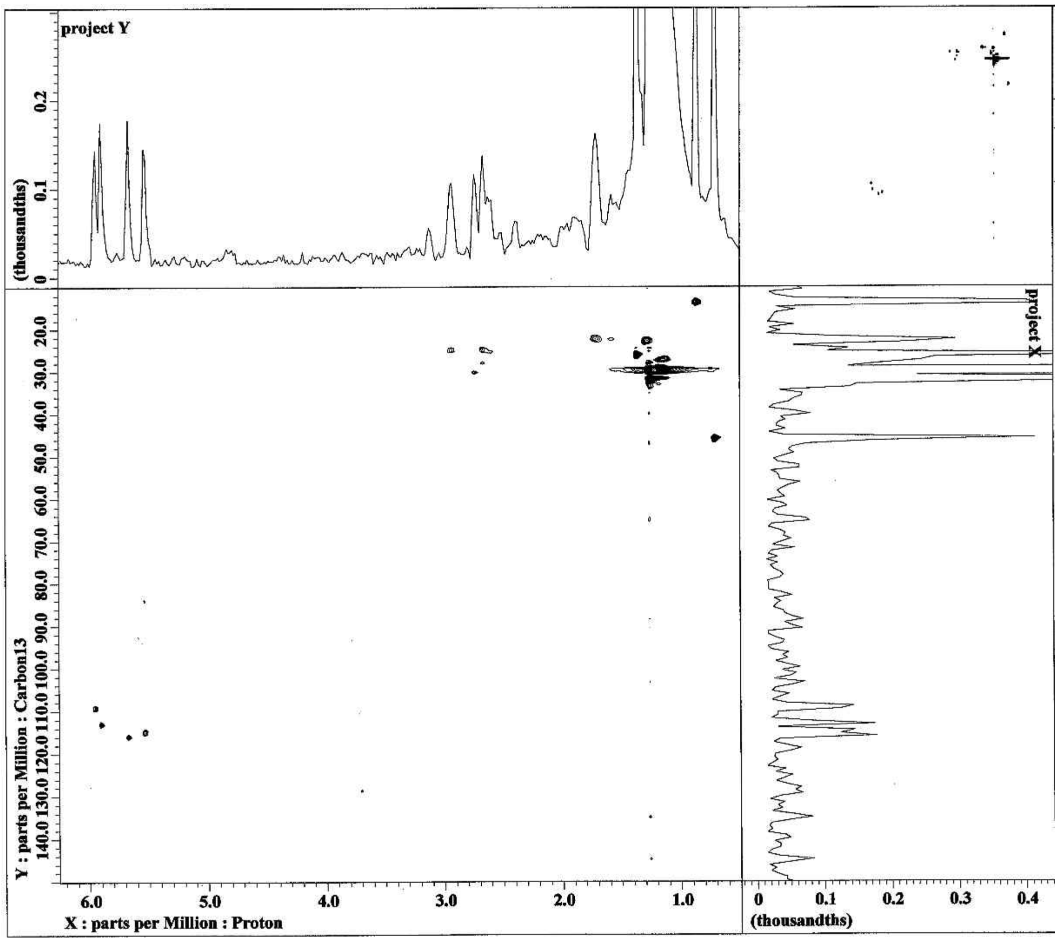

Figure S10. ^{19}F NMR spectrum of [**1**-Zr(Me)(N(Me)($C_{18}H_{37}$)$_2$)]$^+$[B(C_6F_5)$_4$]$^-$.

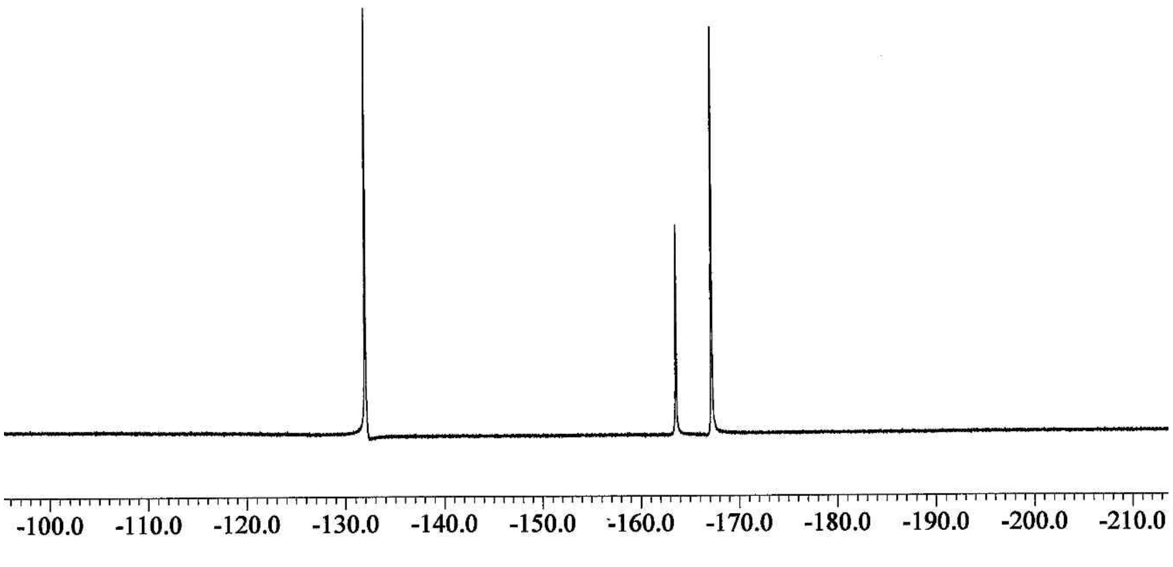

Figure S11. ^{1}H NMR spectra of $Me(C_{18}H_{37})_2N$ and $[(Me)(C_{18}H_{37})_2N\text{-}H]^+[B(C_6F_5)_4]^-$ recorded in C_6D_{12}.

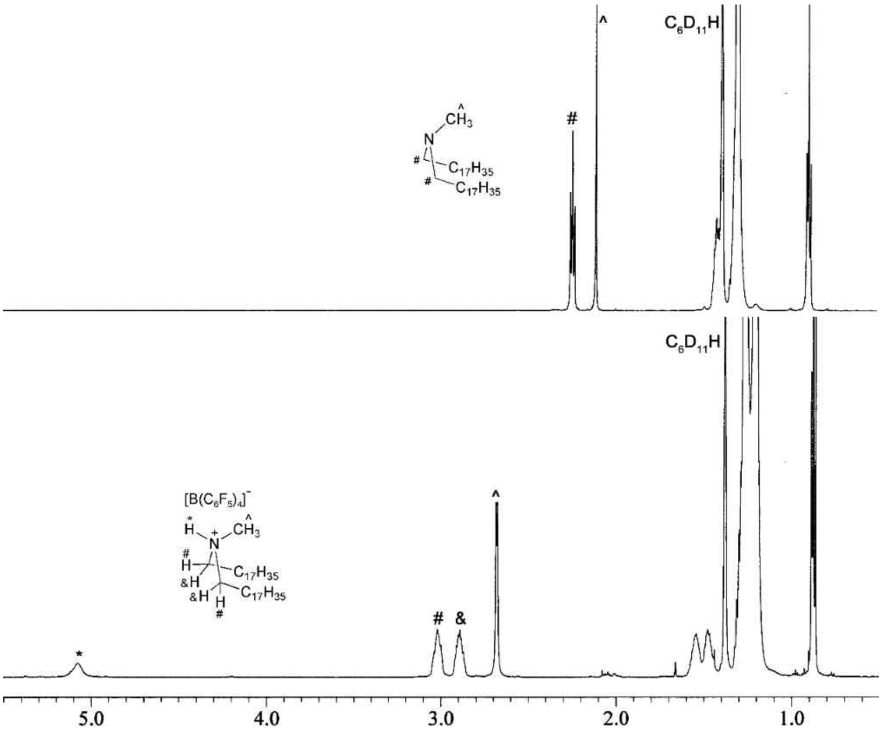

Figure S12. ^{1}H NMR spectra of **4**-$HfMe_2$ and its activated complex $[\mathbf{4}\text{-}Hf(Me)(N(Me)(C_{18}H_{37})_2)]^+[B(C_6F_5)_4]^-$.

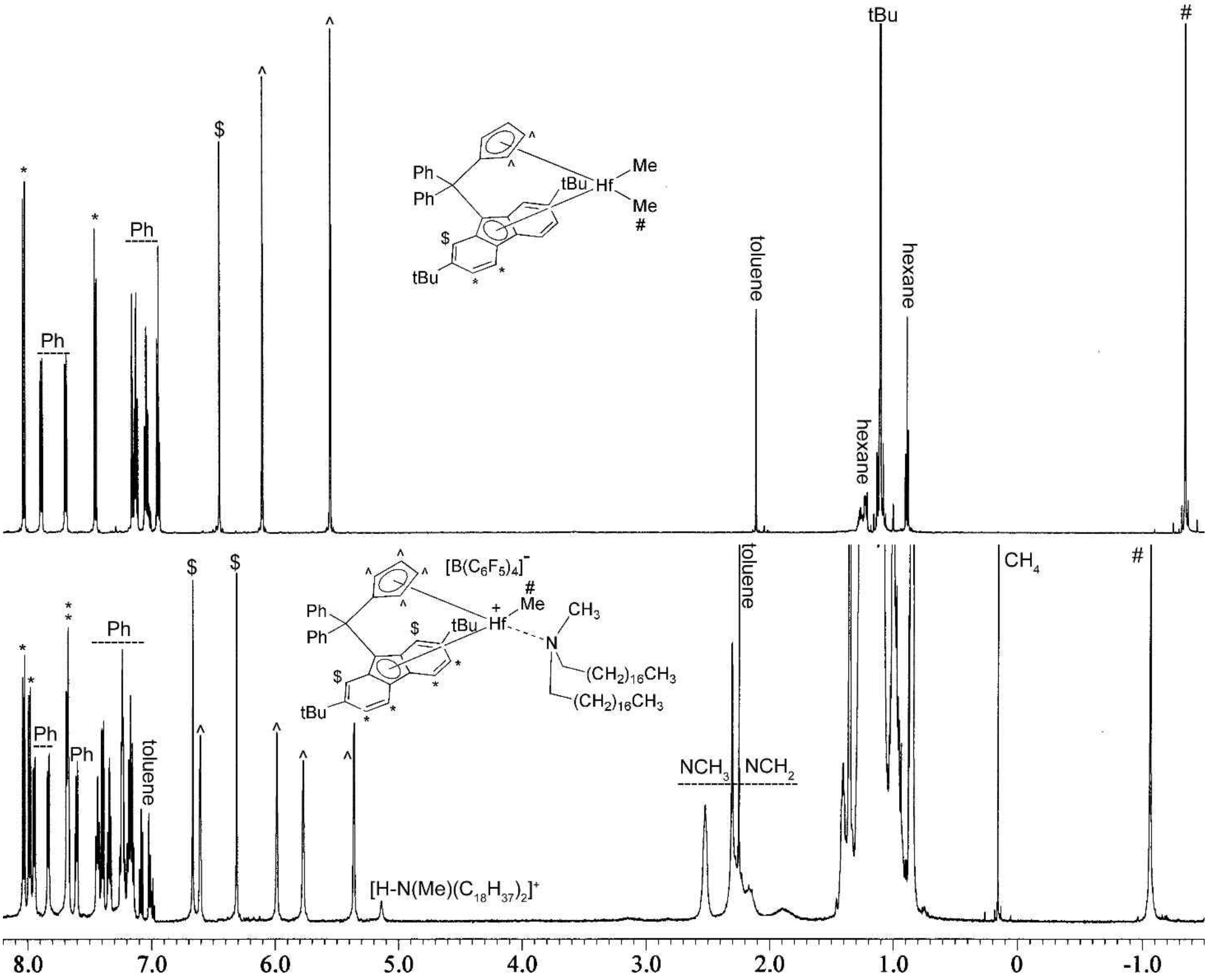

Figure S13. ^{1}H NMR spectra of **5**-TiMe$_2$ (**a**) and its reaction product with $[(Me)(C_{18}H_{37})_2N\text{-}H]^+[B(C_6F_5)_4]^-$ (**b**).

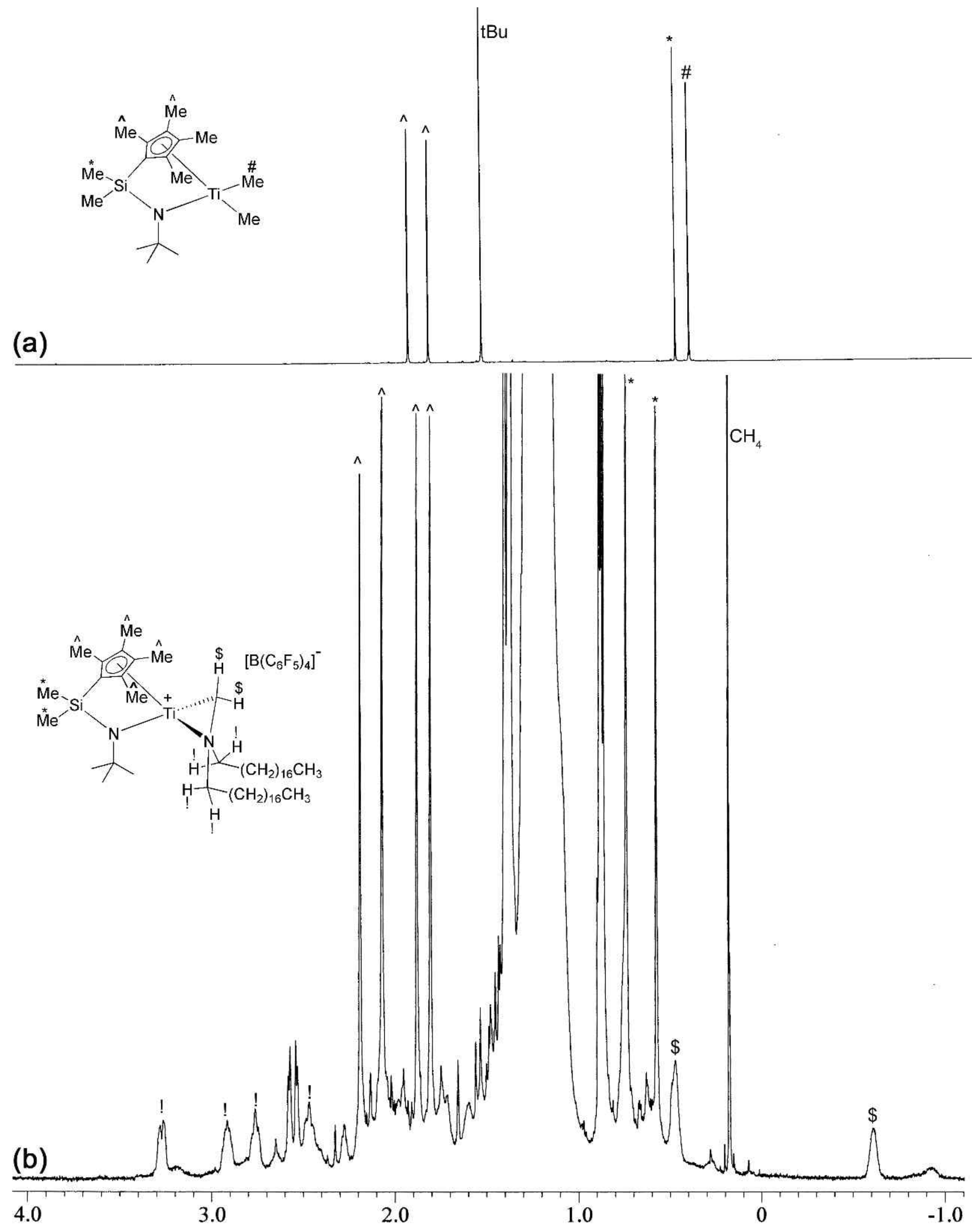

Figure S14. ^{1}H NMR spectra for the reaction of **6**-$TiMe_2$ with $[(C_{18}H_{37})_2NH_2]^+[B(C_6F_5)_4]^-$ in 10 min (**a**) and in 2 h (**b**).

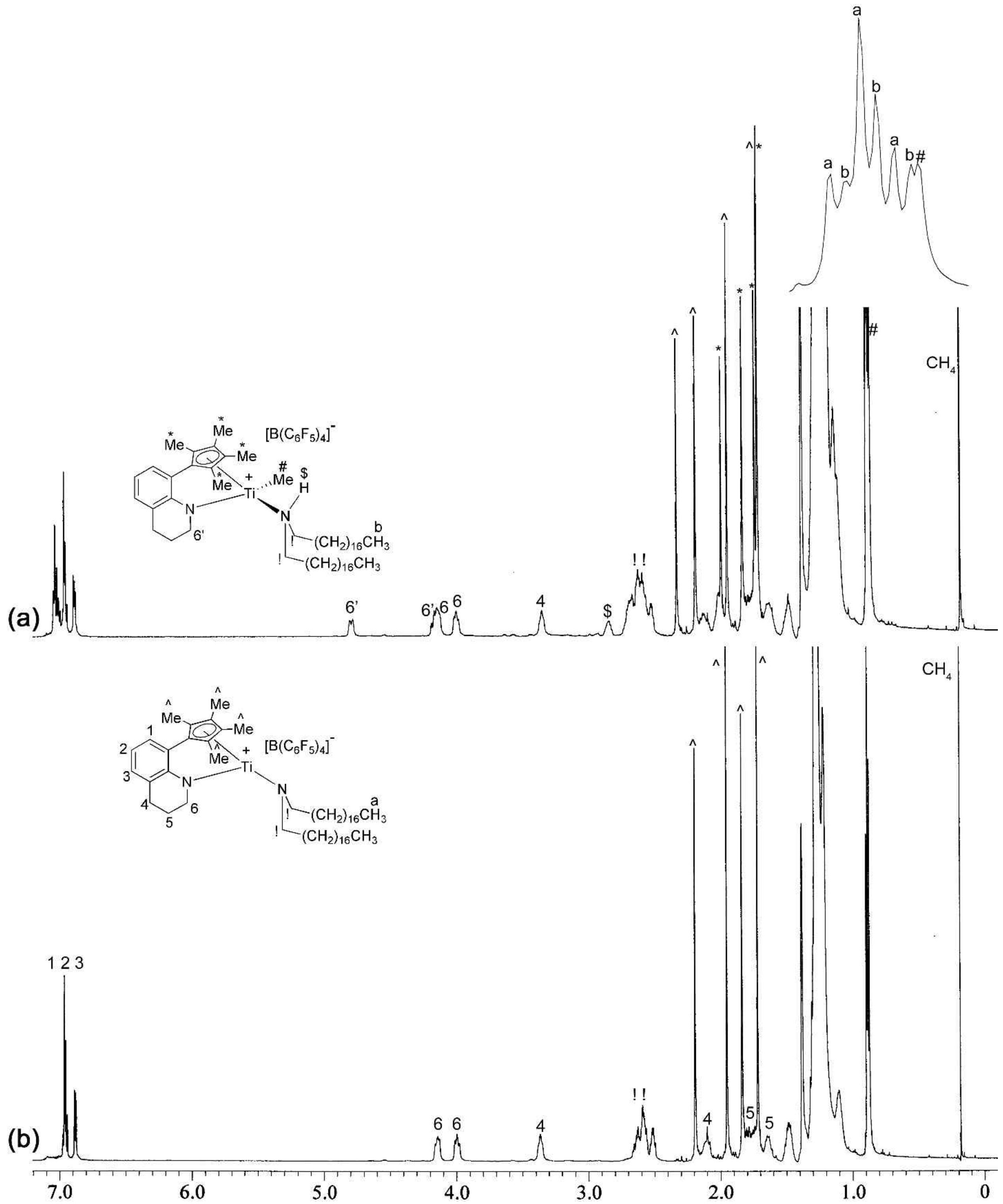

Figure S15. ^{1}H NMR spectra of **7**-TiMe$_2$ (**a**) and its activated complex [**7**-Ti(η^1-CH$_2$)N(C$_{18}$H$_{37}$)$_2$]$^+$[B(C$_6$F$_5$)$_4$]$^-$ formed at an initial stage (**b**) which was transformed to another species after overnight (**c**).

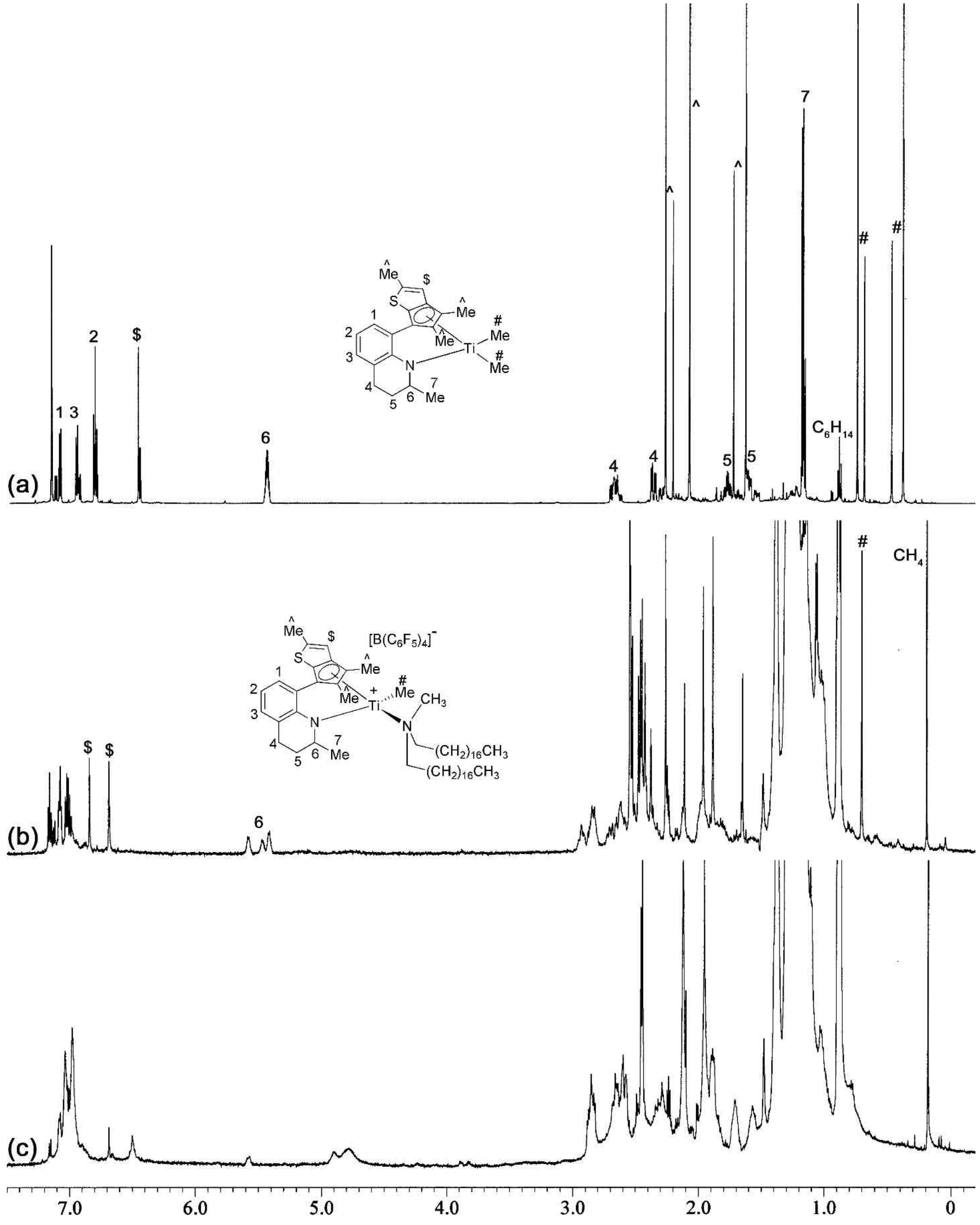

checkCIF/PLATON report

Structure factors have been supplied for datablock(s) lby152_0m

THIS REPORT IS FOR GUIDANCE ONLY. IF USED AS PART OF A REVIEW PROCEDURE FOR PUBLICATION, IT SHOULD NOT REPLACE THE EXPERTISE OF AN EXPERIENCED CRYSTALLOGRAPHIC REFEREE.

No syntax errors found.   CIF dictionary   Interpreting this report

Datablock: lby152_0m

```
Bond precision:  C-C = 0.0084 A          Wavelength=0.71073

Cell:          a=15.1760(2)      b=15.9555(2)     c=17.8025(3)
               alpha=63.5766(6)  beta=69.4527(8)  gamma=81.8613(7)
Temperature:   100 K

                 Calculated                Reported
Volume           3614.23(9)                3614.24(9)
Space group      P -1                      P -1
Hall group       -P 1                      -P 1
Moiety formula   C41 H44 Hf, 0.4(C7 H8)    2(C41 H44 Hf), 0.8(C7 H8)
Sum formula      C43.80 H47.20 Hf          C87.60 H94.40 Hf2
Mr               752.11                    1504.21
Dx,g cm-3        1.382                     1.382
Z                4                         2
Mu (mm-1)        2.914                     2.914
F000             1528.0                    1528.0
F000'            1526.13
h,k,lmax         18,19,21                  18,19,21
Nref             13922                     13827
Tmin,Tmax                                  0.638,0.745
Tmin'

Correction method= # Reported T Limits: Tmin=0.638 Tmax=0.745
AbsCorr = MULTI-SCAN

Data completeness= 0.993          Theta(max)= 25.802

R(reflections)= 0.0400( 10040)    wR2(reflections)= 0.0924( 13827)

S = 1.032                 Npar= 869
```

The following ALERTS were generated. Each ALERT has the format
test-name_ALERT_alert-type_alert-level.
Click on the hyperlinks for more details of the test.

Alert level B

```
PLAT230_ALERT_2_B Hirshfeld Test Diff for    C77      --C78      .       12.5 s.u.
```

Alert level C

```
PLAT053_ALERT_1_C Minimum Crystal Dimension Missing (or Error) ...     Please Check
PLAT054_ALERT_1_C Medium  Crystal Dimension Missing (or Error) ...     Please Check
PLAT055_ALERT_1_C Maximum Crystal Dimension Missing (or Error) ...     Please Check
PLAT213_ALERT_2_C Atom C4              has ADP max/min Ratio .....        3.2 prolat
PLAT220_ALERT_2_C NonSolvent   Resd 1  C   Ueq(max)/Ueq(min) Range        4.1 Ratio
PLAT220_ALERT_2_C NonSolvent   Resd 2  C   Ueq(max)/Ueq(min) Range        5.5 Ratio
PLAT222_ALERT_3_C NonSolvent Resd 1  H   Uiso(max)/Uiso(min) Range        4.5 Ratio
PLAT222_ALERT_3_C NonSolvent Resd 2  H   Uiso(max)/Uiso(min) Range        6.0 Ratio
PLAT234_ALERT_4_C Large Hirshfeld Difference C77      --C80      .       0.16 Ang.
PLAT234_ALERT_4_C Large Hirshfeld Difference C83      --C89      .       0.22 Ang.
PLAT242_ALERT_2_C Low    'MainMol' Ueq as Compared to Neighbors of        C36 Check
PLAT242_ALERT_2_C Low    'MainMol' Ueq as Compared to Neighbors of        C77 Check
PLAT250_ALERT_2_C Large U3/U1 Ratio for Average U(i,j) Tensor ....        3.3 Note
PLAT260_ALERT_2_C Large Average Ueq of Residue Including       C83      0.162 Check
PLAT342_ALERT_3_C Low Bond Precision on  C-C Bonds ...............     0.0084 Ang.
PLAT910_ALERT_3_C Missing # of FCF Reflection(s) Below Theta(Min).          5 Note
PLAT911_ALERT_3_C Missing FCF Refl Between Thmin & STh/L=    0.600         61 Report
PLAT977_ALERT_2_C Check Negative Difference Density on H79A             -0.37 eA-3
```

Alert level G

```
PLAT002_ALERT_2_G Number of Distance or Angle Restraints on AtSite          7 Note
PLAT003_ALERT_2_G Number of Uiso or Uij Restrained non-H Atoms ...         14 Report
PLAT042_ALERT_1_G Calc. and Reported MoietyFormula Strings  Differ     Please Check
PLAT045_ALERT_1_G Calculated and Reported Z Differ by a Factor ...       2.00 Check
PLAT172_ALERT_4_G The CIF-Embedded .res File Contains DFIX Records          3 Report
PLAT173_ALERT_4_G The CIF-Embedded .res File Contains DANG Records          5 Report
PLAT174_ALERT_4_G The CIF-Embedded .res File Contains FLAT Records          1 Report
PLAT176_ALERT_4_G The CIF-Embedded .res File Contains SADI Records          3 Report
PLAT178_ALERT_4_G The CIF-Embedded .res File Contains SIMU Records          1 Report
PLAT186_ALERT_4_G The CIF-Embedded .res File Contains ISOR Records          2 Report
PLAT187_ALERT_4_G The CIF-Embedded .res File Contains RIGU Records          1 Report
PLAT301_ALERT_3_G Main Residue  Disorder .............(Resd  1  )          7% Note
PLAT302_ALERT_4_G Anion/Solvent/Minor-Residue Disorder (Resd  3  )       100% Note
PLAT412_ALERT_2_G Short Intra XH3 .. XHn     H29      ..H38C     .       2.11 Ang.
                                                      x,y,z  =      1_555 Check
PLAT412_ALERT_2_G Short Intra XH3 .. XHn     H31      ..H39D     .       2.13 Ang.
                                                      x,y,z  =      1_555 Check
PLAT790_ALERT_4_G Centre of Gravity not Within Unit Cell: Resd.  #          2 Note
              C41 H44 Hf
PLAT790_ALERT_4_G Centre of Gravity not Within Unit Cell: Resd.  #          3 Note
              C7 H8
PLAT860_ALERT_3_G Number of Least-Squares Restraints .............        178 Note
PLAT883_ALERT_1_G No Info/Value for _atom_sites_solution_primary .     Please Do !
PLAT912_ALERT_4_G Missing # of FCF Reflections Above STh/L=  0.600         29 Note
PLAT933_ALERT_2_G Number of OMIT Records in Embedded .res File ...         54 Note
PLAT941_ALERT_3_G Average HKL Measurement Multiplicity ...........        3.6 Low
PLAT978_ALERT_2_G Number C-C Bonds with Positive Residual Density.          2 Info
```

0 **ALERT level A** = Most likely a serious problem - resolve or explain
1 **ALERT level B** = A potentially serious problem, consider carefully
18 **ALERT level C** = Check. Ensure it is not caused by an omission or oversight
23 **ALERT level G** = General information/check it is not something unexpected

6 ALERT type 1 CIF construction/syntax error, inconsistent or missing data

```
15 ALERT type 2 Indicator that the structure model may be wrong or deficient
 8 ALERT type 3 Indicator that the structure quality may be low
13 ALERT type 4 Improvement, methodology, query or suggestion
 0 ALERT type 5 Informative message, check
```

It is advisable to attempt to resolve as many as possible of the alerts in all categories. Often the minor alerts point to easily fixed oversights, errors and omissions in your CIF or refinement strategy, so attention to these fine details can be worthwhile. In order to resolve some of the more serious problems it may be necessary to carry out additional measurements or structure refinements. However, the purpose of your study may justify the reported deviations and the more serious of these should normally be commented upon in the discussion or experimental section of a paper or in the "special_details" fields of the CIF. checkCIF was carefully designed to identify outliers and unusual parameters, but every test has its limitations and alerts that are not important in a particular case may appear. Conversely, the absence of alerts does not guarantee there are no aspects of the results needing attention. It is up to the individual to critically assess their own results and, if necessary, seek expert advice.

Publication of your CIF in IUCr journals

A basic structural check has been run on your CIF. These basic checks will be run on all CIFs submitted for publication in IUCr journals (*Acta Crystallographica*, *Journal of Applied Crystallography*, *Journal of Synchrotron Radiation*); however, if you intend to submit to *Acta Crystallographica Section C* or *E* or *IUCrData*, you should make sure that full publication checks are run on the final version of your CIF prior to submission.

Publication of your CIF in other journals

Please refer to the *Notes for Authors* of the relevant journal for any special instructions relating to CIF submission.

PLATON version of 05/12/2020; check.def file version of 05/12/2020

Datablock lby152_0m - ellipsoid plot

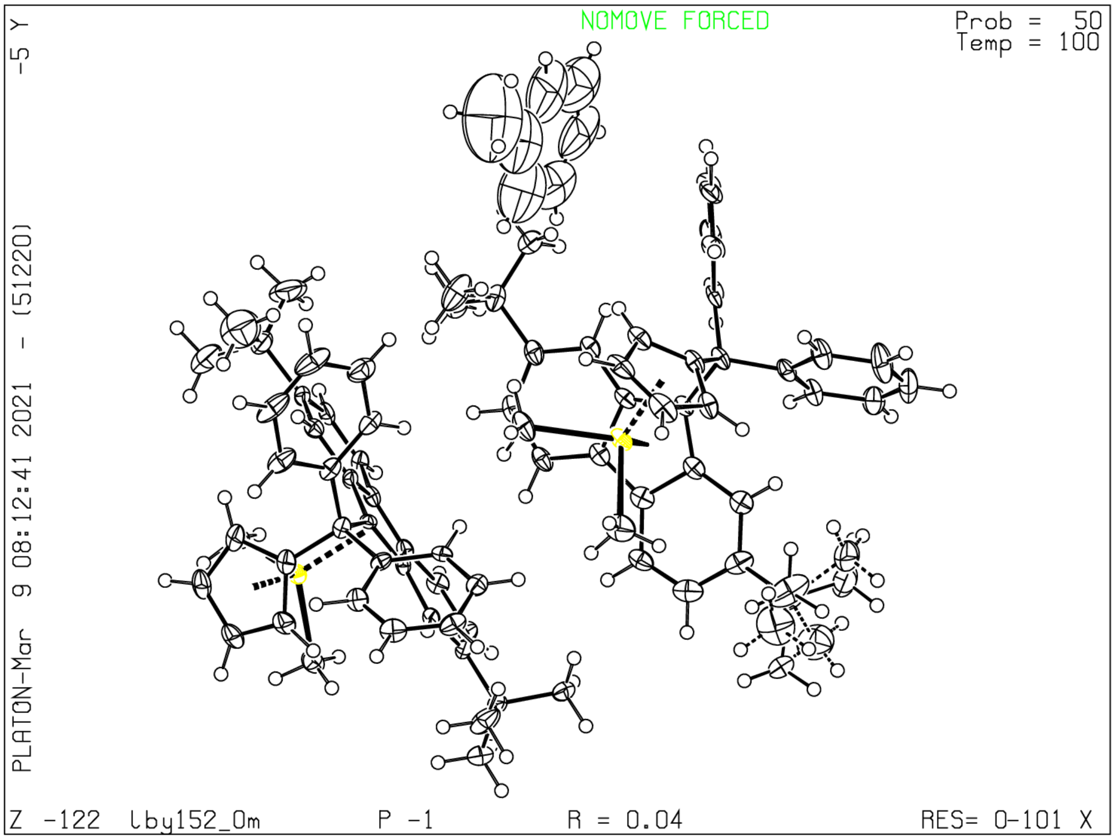

checkCIF/PLATON report

Structure factors have been supplied for datablock(s) lby151

THIS REPORT IS FOR GUIDANCE ONLY. IF USED AS PART OF A REVIEW PROCEDURE FOR PUBLICATION, IT SHOULD NOT REPLACE THE EXPERTISE OF AN EXPERIENCED CRYSTALLOGRAPHIC REFEREE.

No syntax errors found. CIF dictionary Interpreting this report

Datablock: lby151

```
Bond precision:  C-C = 0.0053 A          Wavelength=0.71073

Cell:            a=13.3706(9)     b=12.1586(8)       c=11.5983(8)
                 alpha=90         beta=90.542(2)     gamma=90
Temperature:     100 K

                Calculated                 Reported
Volume          1885.4(2)                  1885.4(2)
Space group     P 21/c                     P 1 21/c 1
Hall group      -P 2ybc                    -P 2ybc
Moiety formula  C16 H30 Cl N Si Ti         C16 H30 Cl N Si Ti
Sum formula     C16 H30 Cl N Si Ti         C16 H30 Cl N Si Ti
Mr              347.82                     347.85
Dx,g cm-3       1.225                      1.225
Z               4                          4
Mu (mm-1)       0.651                      0.651
F000            744.0                      744.0
F000'           746.17
h,k,lmax        16,14,14                   16,14,14
Nref            3586                       3569
Tmin,Tmax                                  0.463,0.745
Tmin'

Correction method= # Reported T Limits: Tmin=0.463 Tmax=0.745
AbsCorr = MULTI-SCAN

Data completeness= 0.995          Theta(max)= 25.716

R(reflections)= 0.0520( 2453)     wR2(reflections)= 0.1479( 3569)

S = 1.070                  Npar= 242
```

The following ALERTS were generated. Each ALERT has the format
test-name_ALERT_alert-type_alert-level.
Click on the hyperlinks for more details of the test.

Alert level B

```
PLAT112_ALERT_2_B ADDSYM Detects New (Pseudo) Symm. Elem         n        100 %Fit
PLAT112_ALERT_2_B ADDSYM Detects New (Pseudo) Symm. Elem         m        100 %Fit
PLAT113_ALERT_2_B ADDSYM Suggests Possible Pseudo/New  Space Group       Pnma Check
```

Alert level C

```
PLAT053_ALERT_1_C Minimum Crystal Dimension Missing (or Error) ...     Please Check
PLAT054_ALERT_1_C Medium  Crystal Dimension Missing (or Error) ...     Please Check
PLAT055_ALERT_1_C Maximum Crystal Dimension Missing (or Error) ...     Please Check
PLAT220_ALERT_2_C NonSolvent   Resd 1  C   Ueq(max)/Ueq(min) Range        5.3 Ratio
PLAT222_ALERT_3_C NonSolvent Resd 1  H   Uiso(max)/Uiso(min) Range        5.3 Ratio
PLAT250_ALERT_2_C Large U3/U1 Ratio for Average U(i,j) Tensor ....        2.2 Note
PLAT906_ALERT_3_C Large K Value in the Analysis of Variance ......      2.047 Check
PLAT911_ALERT_3_C Missing FCF Refl Between Thmin & STh/L=    0.600         12 Report
PLAT977_ALERT_2_C Check Negative Difference Density on H17A             -0.33 eA-3
PLAT977_ALERT_2_C Check Negative Difference Density on H16C             -0.35 eA-3
```

Alert level G

```
PLAT003_ALERT_2_G Number of Uiso or Uij Restrained non-H Atoms ...          5 Report
PLAT186_ALERT_4_G The CIF-Embedded .res File Contains ISOR Records          2 Report
PLAT301_ALERT_3_G Main Residue  Disorder .............(Resd  1  )        25% Note
PLAT413_ALERT_2_G Short Inter XH3 .. XHn     H7A      ..H17A     .       2.13 Ang.
                                              x,3/2-y,1/2+z  =      4_576 Check
PLAT413_ALERT_2_G Short Inter XH3 .. XHn     H8C      ..H16C     .       2.11 Ang.
                                              x,3/2-y,1/2+z  =      4_576 Check
PLAT779_ALERT_4_G Suspect or Irrelevant (Bond) Angle(s) in CIF . #         52 Check
              N1   -SI1  -TI1     1.555   1.555   1.555          39.84 Deg.
PLAT860_ALERT_3_G Number of Least-Squares Restraints ............          30 Note
PLAT912_ALERT_4_G Missing # of FCF Reflections Above STh/L=  0.600          6 Note
PLAT913_ALERT_3_G Missing # of Very Strong Reflections in FCF ....          1 Note
PLAT933_ALERT_2_G Number of OMIT Records in Embedded .res File ...          9 Note
PLAT978_ALERT_2_G Number C-C Bonds with Positive Residual Density.          6 Info
```

0 **ALERT level A** = Most likely a serious problem - resolve or explain
3 **ALERT level B** = A potentially serious problem, consider carefully
10 **ALERT level C** = Check. Ensure it is not caused by an omission or oversight
11 **ALERT level G** = General information/check it is not something unexpected

3 ALERT type 1 CIF construction/syntax error, inconsistent or missing data
12 ALERT type 2 Indicator that the structure model may be wrong or deficient
6 ALERT type 3 Indicator that the structure quality may be low
3 ALERT type 4 Improvement, methodology, query or suggestion
0 ALERT type 5 Informative message, check

It is advisable to attempt to resolve as many as possible of the alerts in all categories. Often the minor alerts point to easily fixed oversights, errors and omissions in your CIF or refinement strategy, so attention to these fine details can be worthwhile. In order to resolve some of the more serious problems it may be necessary to carry out additional measurements or structure refinements. However, the purpose of your study may justify the reported deviations and the more serious of these should normally be commented upon in the discussion or experimental section of a paper or in the "special_details" fields of the CIF. checkCIF was carefully designed to identify outliers and unusual parameters, but every test has its limitations and alerts that are not important in a particular case may appear. Conversely, the absence of alerts does not guarantee there are no aspects of the results needing attention. It is up to the individual to critically assess their own results and, if necessary, seek expert advice.

Publication of your CIF in IUCr journals

A basic structural check has been run on your CIF. These basic checks will be run on all CIFs submitted for publication in IUCr journals (*Acta Crystallographica*, *Journal of Applied Crystallography*, *Journal of Synchrotron Radiation*); however, if you intend to submit to *Acta Crystallographica Section C* or *E* or *IUCrData*, you should make sure that full publication checks are run on the final version of your CIF prior to submission.

Publication of your CIF in other journals

Please refer to the *Notes for Authors* of the relevant journal for any special instructions relating to CIF submission.

PLATON version of 22/03/2021; check.def file version of 19/03/2021

Datablock lby151 - ellipsoid plot

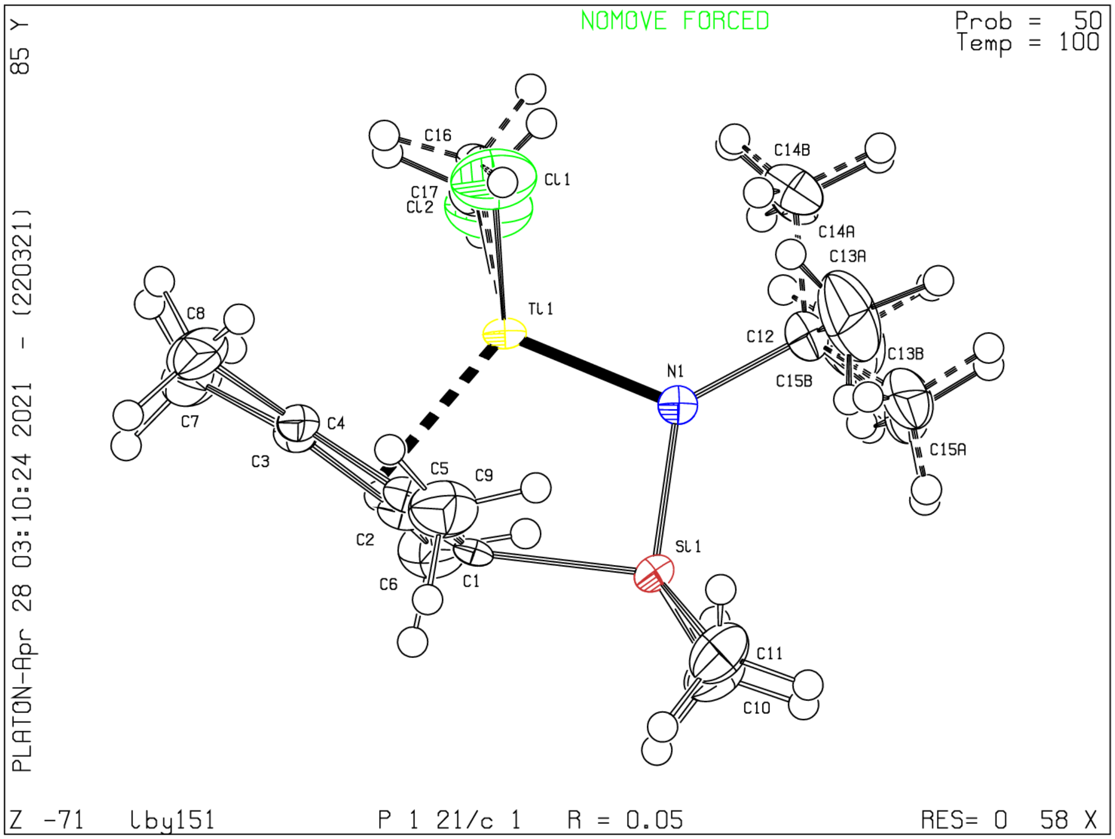

checkCIF/PLATON report

Structure factors have been supplied for datablock(s) lby150-1

THIS REPORT IS FOR GUIDANCE ONLY. IF USED AS PART OF A REVIEW PROCEDURE FOR PUBLICATION, IT SHOULD NOT REPLACE THE EXPERTISE OF AN EXPERIENCED CRYSTALLOGRAPHIC REFEREE.

No syntax errors found. CIF dictionary Interpreting this report

Datablock: lby150-1

```
Bond precision:  C-C = 0.0059 A          Wavelength=0.71073

Cell:            a=13.3739(3)     b=17.6098(3)     c=14.9636(3)
                 alpha=90         beta=90          gamma=90
Temperature:     100 K

                Calculated                 Reported
Volume          3524.10(12)                3524.10(12)
Space group     P b c a                    P b c a
Hall group      -P 2ac 2ab                 -P 2ac 2ab
Moiety formula  C19 H24 Cl N Ti            C19 H24 Cl N Ti
Sum formula     C19 H24 Cl N Ti            C19 H24 Cl N Ti
Mr              349.71                     349.71
Dx,g cm-3       1.318                      1.318
Z               8                          8
Mu (mm-1)       0.634                      0.634
F000            1472.0                     1472.0
F000'           1475.78
h,k,lmax        16,21,18                   16,21,18
Nref            3365                       3348
Tmin,Tmax                                  0.630,0.745
Tmin'

Correction method= # Reported T Limits: Tmin=0.630 Tmax=0.745
AbsCorr = MULTI-SCAN

Data completeness= 0.995          Theta(max)= 25.725

R(reflections)= 0.0587( 1803)     wR2(reflections)= 0.1519( 3348)

S = 1.031                 Npar= 224
```

The following ALERTS were generated. Each ALERT has the format
test-name_ALERT_alert-type_alert-level.
Click on the hyperlinks for more details of the test.

Alert level B

```
RINTA01_ALERT_3_B  The value of Rint is greater than 0.18
            Rint given   0.180
```

Alert level C

```
PLAT053_ALERT_1_C Minimum Crystal Dimension Missing (or Error) ...     Please Check
PLAT054_ALERT_1_C Medium  Crystal Dimension Missing (or Error) ...     Please Check
PLAT055_ALERT_1_C Maximum Crystal Dimension Missing (or Error) ...     Please Check
PLAT906_ALERT_3_C Large K Value in the Analysis of Variance ......      3.903 Check
PLAT911_ALERT_3_C Missing FCF Refl Between Thmin & STh/L=    0.600          7 Report
```

Alert level G

```
PLAT003_ALERT_2_G Number of Uiso or Uij Restrained non-H Atoms ...          2 Report
PLAT020_ALERT_3_G The Value of Rint is Greater Than 0.12 .........      0.180 Report
PLAT186_ALERT_4_G The CIF-Embedded .res File Contains ISOR Records          1 Report
PLAT301_ALERT_3_G Main Residue  Disorder ..............(Resd  1  )         9% Note
PLAT860_ALERT_3_G Number of Least-Squares Restraints .............         12 Note
PLAT910_ALERT_3_G Missing # of FCF Reflection(s) Below Theta(Min).          3 Note
PLAT912_ALERT_4_G Missing # of FCF Reflections Above STh/L=  0.600          8 Note
PLAT933_ALERT_2_G Number of OMIT Records in Embedded .res File ...         10 Note
PLAT978_ALERT_2_G Number C-C Bonds with Positive Residual Density.          1 Info
```

0 **ALERT level A** = Most likely a serious problem - resolve or explain
1 **ALERT level B** = A potentially serious problem, consider carefully
5 **ALERT level C** = Check. Ensure it is not caused by an omission or oversight
9 **ALERT level G** = General information/check it is not something unexpected

3 ALERT type 1 CIF construction/syntax error, inconsistent or missing data
3 ALERT type 2 Indicator that the structure model may be wrong or deficient
7 ALERT type 3 Indicator that the structure quality may be low
2 ALERT type 4 Improvement, methodology, query or suggestion
0 ALERT type 5 Informative message, check

It is advisable to attempt to resolve as many as possible of the alerts in all categories. Often the minor alerts point to easily fixed oversights, errors and omissions in your CIF or refinement strategy, so attention to these fine details can be worthwhile. In order to resolve some of the more serious problems it may be necessary to carry out additional measurements or structure refinements. However, the purpose of your study may justify the reported deviations and the more serious of these should normally be commented upon in the discussion or experimental section of a paper or in the "special_details" fields of the CIF. checkCIF was carefully designed to identify outliers and unusual parameters, but every test has its limitations and alerts that are not important in a particular case may appear. Conversely, the absence of alerts does not guarantee there are no aspects of the results needing attention. It is up to the individual to critically assess their own results and, if necessary, seek expert advice.

Publication of your CIF in IUCr journals

A basic structural check has been run on your CIF. These basic checks will be run on all CIFs submitted for publication in IUCr journals (*Acta Crystallographica*, *Journal of Applied Crystallography*, *Journal of Synchrotron Radiation*); however, if you intend to submit to *Acta Crystallographica Section C* or *E* or *IUCrData*, you should make sure that full publication checks are run on the final version of your CIF prior to submission.

Publication of your CIF in other journals

Please refer to the *Notes for Authors* of the relevant journal for any special instructions relating to CIF submission.

PLATON version of 22/03/2021; check.def file version of 19/03/2021

Datablock lby150-1 - ellipsoid plot

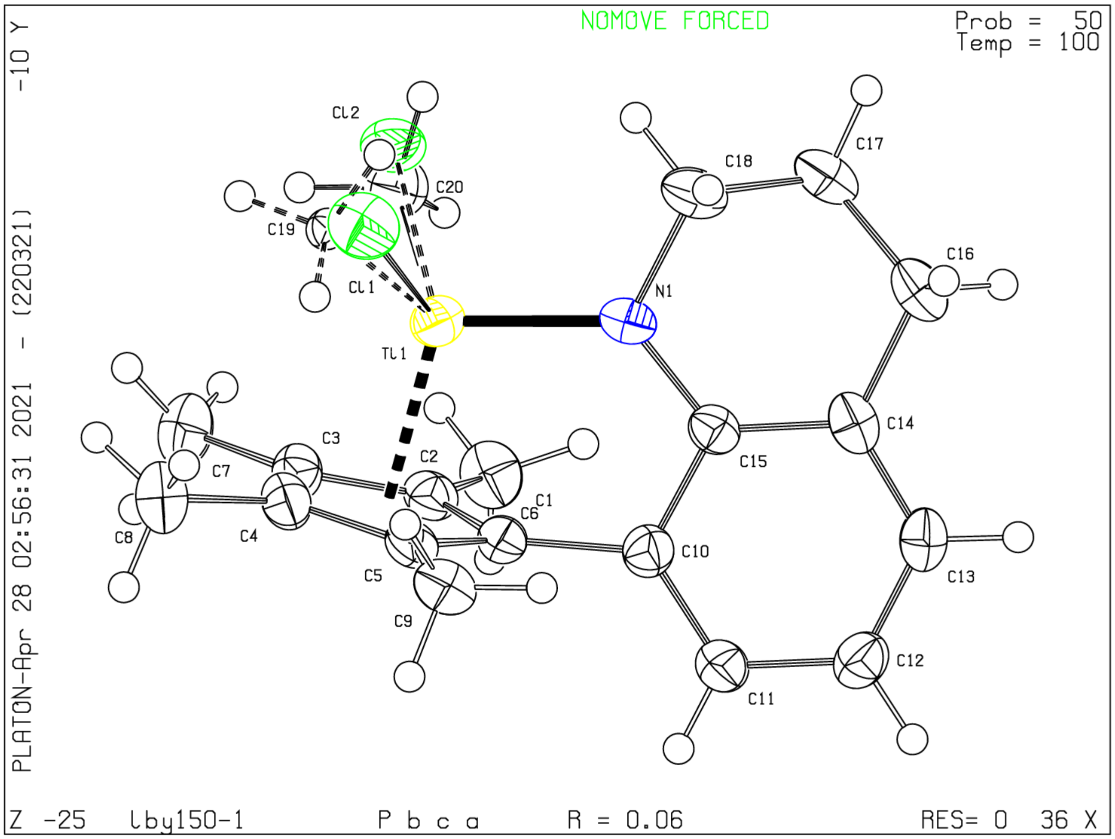

Supplement: Supplementary file 1 [file molecules-26-02827-s001.zip › molecules-1198118-supplementary.pdf]
